# Additives for Aluminum‐Air Batteries: A Review

**DOI:** 10.1002/smll.202514913

**Published:** 2026-01-31

**Authors:** Hajar Mahmoudi, Asrar Alam, Patrick Theato, Maria Felicia Gaele, Pasquale Gargiulo, Huijing Li, Tonia Mariarosaria Di Palma

**Affiliations:** ^1^ Consiglio Nazionale delle Ricerche (CNR)‐Istituto di Scienze e Tecnologie per l'Energia e la Mobilità Sostenibili (STEMS) Naples Italy; ^2^ Wallenberg Initiative Materials Science for Sustainability (WISE) Department of Fibre and Polymer Technology School of Engineering Sciences in Chemistry KTH Royal Institute of Technology Stockholm Sweden; ^3^ Karlsruhe Institute of Technology (KIT) Institute for Chemical Technology and Polymer Chemistry Karlsruhe Germany; ^4^ Karlsruhe Institute of Technology (KIT) Soft Matter Synthesis Laboratory Institute for Biological Interfaces III Eggenstein‐Leopoldshafen Germany; ^5^ Dipartimento di Ingegneria Chimica dei Materiali e della Produzione Industriale Università degli Studi di Napoli Federico II Naples Italy

**Keywords:** aluminum air battery, hybrid additive, inorganic additive, organic additive

## Abstract

The growing demand for efficient energy storage systems directs substantial research attention toward aluminum–air batteries, primarily due to their low cost and the abundant availability of aluminum. Among the various strategies aimed at enhancing their performance, the incorporation of electrolyte additives emerges as one of the most cost‐effective and efficient approaches. Electrolyte additives, usually constituting approximately 1% of the total electrolyte composition, actively influence the physicochemical characteristics of both the electrolyte and the electrode–electrolyte interface, thereby contributing to marked enhancements in the overall performance of aluminum–air batteries. Despite their low concentrations, additives play a fundamental role in enhancing the efficiency and extending the service life of aluminum–air batteries by stabilizing the electrode–electrolyte interface and promoting favorable electrochemical performance. This review investigates the primary factors propelling the advancement of aluminum–air batteries by considering the diverse functions of electrolyte additives. The additives are classified into three categories: organic, inorganic, and hybrid. This comprehensive analysis aims to serve as a key resource for the informed selection and development of electrolyte additives, thereby fostering continued innovation in aluminum–air battery technologies.

## Graphical abstract

The role of electrolyte additives in Al–air batteries is investigated by focusing on their capacity to moderate Al corrosion rates, inhibit self‐discharge, and prevent surface barrier formation. Additives present in organic, inorganic, and hybrid categories, which regulate the Al–electrolyte interface by forming protective films on the Al surface, limit water accessibility at the Al interface, and limit Al anode corrosion.

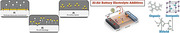

## 1 Introduction

Lithium‐ion batteries (LIBs) remain the most technologically advanced and extensively utilized systems for electrochemical energy storage and conversion [1, 2, 3, 4, 5]. Despite their commercial success, the large‐scale deployment of LIBs is increasingly limited by intrinsic drawbacks, including safety concerns, complex end‐of‐life recycling and disposal, the finite and geopolitically concentrated availability of lithium, high manufacturing costs, and considerable environmental ramifications related to lithium as well as components of utilized cathode materials such as cobalt [6, 7, 8]. These constraints have stimulated intensive research efforts aimed at developing next‐generation “post‐lithium” battery technologies that offer enhanced sustainability, economic viability, and electrochemical performance, while satisfying essential criteria such as compactness, low weight, operational reliability, and safety [9, 10].

Among the many alternative battery technologies, aluminum‐based batteries are currently investigated as particularly promising candidates for replacing conventional lithium‐based systems [11, 12, 13, 14]. This stems from the intrinsic properties of metallic aluminum (Al), namely its high volumetric capacity (>8000 mAh mL^−^^1^) and gravimetric capacity (2.98 Ah g^−^^1^, ∼77% of that of Li) [15]. These characteristics could endow Al‐based electrochemical systems with markedly higher theoretical specific capacities and energy densities.

Al‐based batteries are primarily distinguished by their electrolyte composition and the mechanisms governing ion transport and redox reactions. These systems can be broadly divided into aqueous and non‐aqueous categories [16]. Aqueous systems, such as Al–air and aqueous Al–ion batteries, utilize water‐based electrolytes that facilitate rapid ionic conduction, offering high conductivity and ease of ion transport. However, the limited electrochemical stability of water and its narrow voltage window restrict the achievable energy density and impose challenges for long‐term cycling. In contrast, non‐aqueous systems, including Al–graphite and non‐aqueous Al–air batteries, employ ionic liquids or organic solvents as electrolytes, which expand the operational voltage window and enable compatibility with insertion‐type cathodes. These systems can achieve moderate energy densities and improved electrochemical performance, albeit typically at the cost of increased material expense and more complex fabrication processes.

To provide a systematic comparison, Table 1 presents a concise overview of representative Al–air and Al–ion battery chemistries. For the most extensively studied configurations, the table details the key features of the batteries such as (i) the anode and cathode materials, reflecting the fundamental redox reactions and mechanisms that govern cell operation; (ii) the electrolyte composition; (iii) the rechargeability of the system, distinguishing between primary (non‐rechargeable) and secondary (rechargeable) configurations; and (iv) indicative ranges of specific capacity, energy density, and power/rate capability, as reported in contemporary literature.

**TABLE 1:** The key features of Al batteries. The specific capacities for Al–air cells are generally reported per gram of Al consumed (mAh g^−^^1^_Al), whereas for Al–ion batteries they are expressed per gram of active cathode material (mAh g^−^^1^_cathode).

System (macro‐type)	Anode	Cathode	Electrolyte	Typical specific capacity	Typical power density	Reference
Aqueous Al–air batteries (primary)	Al metal or Al alloys	C‐based porous	Aqueous + KOH or NaCl	≈1000–2500 mAh g^−^^1^	≈20–50 mW cm^−^^2^ (classic cells); up to ≈350–545 mW cm^−^^2^ (flow cells), 270 Wh Kg^−1^ (considering the total weight of the systems)	[17, 18, 19, 20]
Non‐aqueous Al–air batteries (primary)	Al metal	C‐based porous	Ionic liquids or organic solvents	up to ≈70% Al utilization	≈1–1.5 mW cm^−^^2^	[16, 21]
Gel Al–air batteries (primary or secondary‐few cycles)	Al metal	C‐based porous	polymer gels (PVA‐based gels in acidic, neutral or alkaline media)	≈1000–2500 mAh g^−^^1^	≈2–20 mW cm^−^^2^	[15, 22, 23]
Non‐aqueous Al–ion batteries (secondary)	Al metal	Graphite	Ionic liquids	≈70–110 mAh g^−^^1^	≈68–70 Wh kg^−^^1^ (including electrodes and electrolyte)	[24, 25, 26]
Aqueous Al–ion batteries (secondary)	Al metal	Mn oxide	High salt concentrated aqueous	≈450–520 mAh g^−^^1^	≈400–500 Wh kg^−^^1^ (at 30–200 mA g^−^^1^)	[27, 28, 29]

Although the values reported in the Table 1 are not intended as a strictly normalized benchmark, since they originate from cells tested under different configurations and conditions, they highlight key trends: i) aqueous Al–air systems provide very high specific capacities, especially if aqueous alkaline electrolyte are used, but are mostly primary, ii) non‐aqueous and aqueous Al–ion chemistries enable true rechargeability with moderate capacities, and iii) rechargeable gel/quasi–solid‐state Al–air cells present slightly lower power density compared to aqueous Al–air cells. Al–air cells currently bridge these regimes, albeit with still limited cycle life. At the present stage of development, aqueous Al–air batteries, and in particular alkaline systems, are generally considered more practical for near‐term applications, owing to their use of inexpensive and abundant components, relatively high‐power density, and simple cell design [30]. They are therefore mainly envisaged as low‐cost primary batteries for backup power, off‐grid and remote energy supply, and other scenarios where high specific energy is valuable, and cell replacement or “mechanical recharge” (anode replacement) is acceptable. In contrast, non‐aqueous Al–air batteries based on organic or ionic liquid electrolytes are still at an exploratory research stage and are primarily investigated to mitigate corrosion and to improve reversibility; as such, they are currently more suited to niche or specialized applications in which wider electrochemical stability windows or improved shelf life may justify higher electrolyte costs and lower technological maturity.

Al–air batteries can achieve higher energy densities due to intrinsically favorable thermodynamics and high potential of the couple Al–O_2,_ which gives a theoretical energy density of approximately 8100 Wh kg^−1^, second only to lithium‐air batteries, which can achieve values approaching 13000 Whkg^−1^ [31]. In practice, Al corrosion and parasitic reactions can markedly reduce the attainable energy. Actually, the corrosion is a pervasive challenge across all Al‐based battery chemistries [32], critically influencing both electrochemical performance and operational durability. However, this problem is especially severe in aqueous alkaline Al–air systems, where aggressive self‐corrosion and parasitic reactions can lead to substantial reductions in cell efficiency, energy output, and cycle life. Such deleterious side reactions undermine the inherent advantages of Al–air batteries as the most promising post‐lithium energy storage and conversion technologies.

**FIGURE 1:**
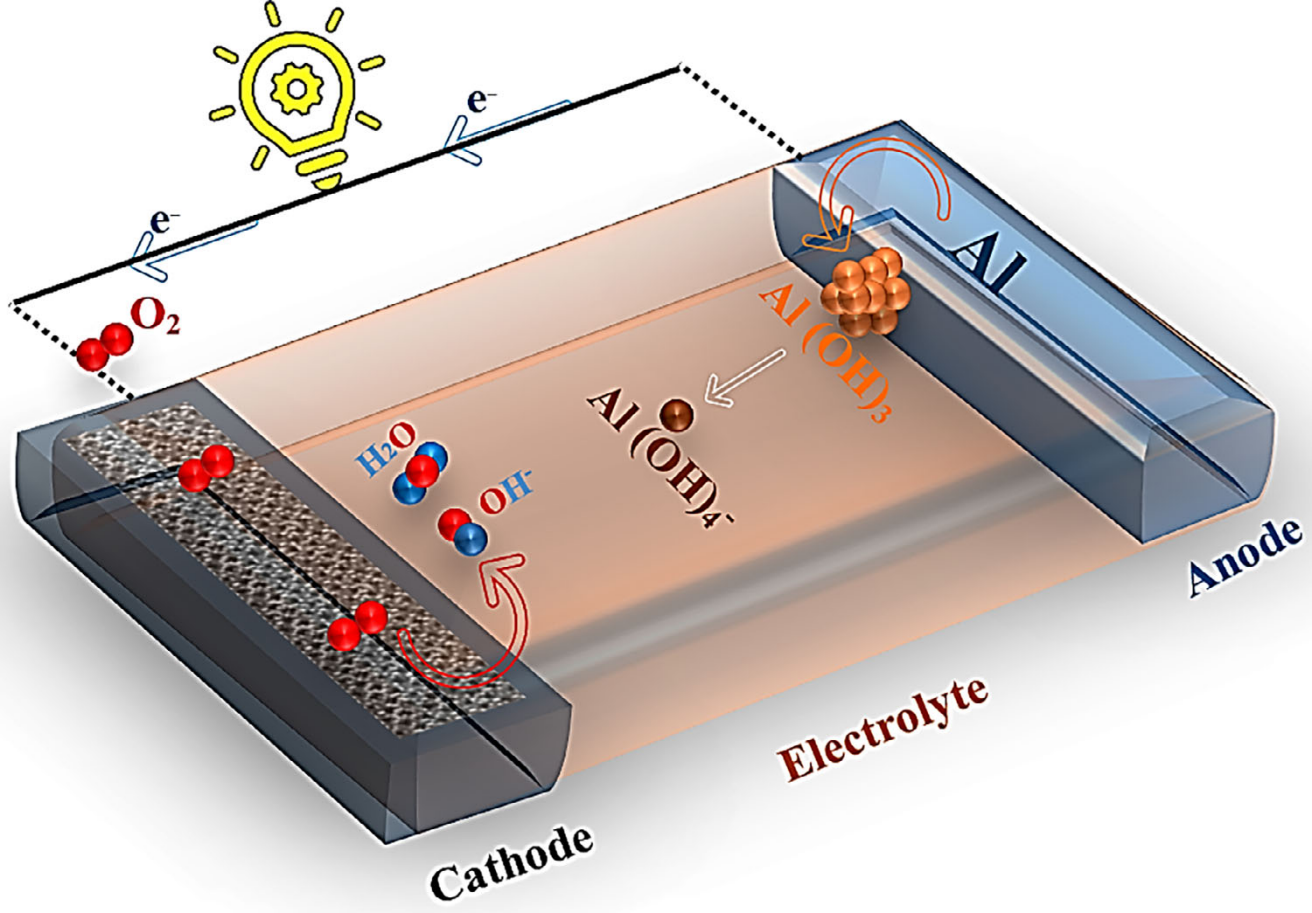
Al–air battery set up.

A typical Al–air cell consists of an Al metal anode, an aqueous or non‐aqueous electrolyte, and a porous carbon‐based air cathode that catalyzes oxygen reduction. During operation, Al is oxidized at the anode, while atmospheric O_2_ is reduced at cathode active sites (Figure 1). The reaction mechanism depends on the pH of the electrolyte [33, 34]. The electrolyte is a crucial component in battery systems, enabling ionic transport between electrodes and significantly influencing electrochemical kinetics and overall cell performance. Aqueous electrolytes are the most widely utilized in Al–air batteries due to their high ionic conductivity, higher specific capacity and power density, low cost, and more environmental benignity [35] and can be used at different pH [36]. By contrast, non‐aqueous electrolytes, including room‐temperature ionic liquids, deep eutectic solvents, and various organic solvents [37], present significant drawbacks such as flammability, toxicity, and environmental concerns [38], and can entail high production costs, particularly when large‐scale deployment is considered [39]. However, aqueous electrolytes also present significant challenges: (i) aqueous Al–air cells are inherently non‐rechargeable because water reduction at the Al anode impedes Al redeposition for charging; (ii) water evaporation; (iii) risk of corrosive electrolyte leakage; (iv) cathode flooding; and (v) spontaneous Al self‐corrosion; (vi) passivation layer at pH range 4–8, which can limit performance [40]. The half‐cell reactions, the theoretical and operating potential, and the self‐corrosion reactions of the Al–air battery through the discharge process are shown in Table 2 [41].

**TABLE 2:** The overall and the self‐corrosion reactions [22].

	Overall reactions	Theorical potentials	Operating potentials	Self‐corrosion reactions
Alkaline media	(1) Al+ 4OH^−^ → Al (OH)_4_^−^ + 3e^−^ (2) 3/4O_2_ +3/2H_2_O+3e^−^→ 3OH^−^	2.33 V 0.40 V		Al + 3H_2_O + OH^−^ → Al(OH)_4_^−^ + 3/2H_2_↑
	Al + 3/4O_2_ +OH^−^+3/2H_2_O → Al(OH)_4_^−^	2.73 V	1.3 V	
Acidic media	(3) Al → Al^3+^ + 3e^−^ (4) 3/4O_2_ +3H^+^+3e^−^ → 3/2H_2_O	1.66 V 1.23 V		Al + 3H^+^ → Al^3+^ + 3/2H_2_↑
	Al + 3/4O_2_ +3H^+^ → Al^3+^ + 3/2H_2_O	2.89 V	1 V	
Neutral media	(5) Al+ 3OH^−^ → Al (OH)_3_ + 3e^−^ (6) 3/4O_2_ +3/2H_2_O+3e^−^→ 3OH^−^	2.31 V 0.40 V		Al + 3H_2_O + OH^−^ → Al(OH)_4_^−^ + 3/2H_2_↑
	Al + 3/4O_2_ +3H_2_O → Al(OH)_3_	2.71 V	0.8 V	

At any pH value, the desired anodic dissolution of Al that sustains the external current occurs in parallel with parasitic self‐corrosion pathways that evolve H_2_, and their relative contributions (i.e. evaluated through anodic efficiency measurements) can change markedly with operating conditions [42]. From the table, it results that the higher theoretical and operating potential are obtained in alkaline Al–air cells. Al(OH)_3_, which is formed as an intermediate in alkaline media and also as the main product in neutral media, is insoluble in water but can dissolve in a dilute strong alkali solution, where it reacts with OH^−^ to form water soluble Al(OH)_4_^−^ [43]. Al(OH)_4_^−^ is formed both during the intended electrochemical oxidation of Al and through parasitic self‐corrosion. When its concentration becomes sufficiently high, it can precipitate as Al(OH)_3_, forming deposits that passivate the anode surface and clog the electrolyte and porous pathways in the cathodes. As a result, cell performance deteriorates, and the battery may eventually fail. At any pH, the chemical self‐corrosion reaction diverts the electrochemical oxidation reaction of Al to the evolution of H_2_, reducing anode utilization, efficiency, and operating voltage compared to theoretical values (Table 2) [37].

To mitigate the persistent challenge of Al self‐corrosion in aqueous electrolytes, two principal strategies have been widely explored: anode modification and electrolyte optimization. First, anode modification focuses on reducing impurity levels in Al or employing specially designed Al alloys containing elements such as Mn, Mg, Sn, Ga, Zn, or In [44]. These alloying elements modify the anode's microstructure and electrochemical behavior, thereby enhancing corrosion resistance and improving battery performance. Second, electrolyte engineering has advanced through four major approaches; (i) gel and solid polymer electrolytes inherently suppress self‐corrosion due to their reduced water content and the physical barrier provided by the polymer matrix [15, 45, 46, 47]. (ii) high‐concentration or “water‐in‐salt” electrolytes reduce hydrogen evolution by confining water molecules within the solvation shells of salt ions. This coordination limits the electrochemical activity of water, thereby restraining parasitic side reactions [34, 37, 48, 49]. (iii) make use of a multi‐electrolyte configuration, such as organic//aqueous or acid//alkaline configurations, establish separate electrochemical environments at each electrode [50, 51]. These systems can increase output voltage and mitigate Al self‐corrosion when an organic electrolyte is on the anode side [52]. (iv) inhibitive additives function by decreasing hydrogen evolution and metal dissolution without compromising cell performance. The addition of corrosion‐inhibiting additives further enhances their protective capabilities by stabilizing the Al‐electrolyte interface [53, 54, 55, 56, 57]. Collectively, these strategies offer effective pathways to improve the durability, efficiency, and practical deployment of Al–air batteries.

This review addresses strategies to mitigate anode corrosion in aqueous Al–air batteries, with a particular focus on the incorporation of electrolyte additives. The literature is systematically surveyed to highlight the chemical composition of various additives, the main mechanisms through which they inhibit corrosion, and quantitative assessments of their effectiveness in improving both anode stability and overall electrochemical performance.

## 2 Corrosion Mitigating Additives in Electrolytes for Al–Air Batteries

**FIGURE 2:**
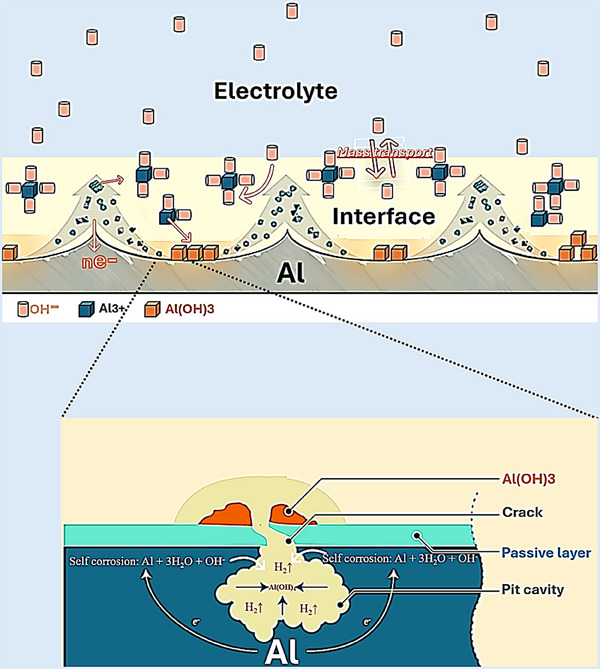
Scheme of corrosion mechanism of Al in aqueos alkaline electrolyte.

In aqueous Al–air batteries, the high chemical reactivity of Al makes the metal intrinsically susceptible to simultaneous anodic dissolution and parasitic hydrogen evolution [58]. These reactions are tightly coupled to the continuous formation, breakdown, and reformation of surface oxide layers. In particular, under strongly alkaline conditions, the native passive film, composed mainly of Al_2_O_3_ and Al(OH)_3_, undergoes persistent cycles of dissolution and reconstruction, producing a highly dynamic, spatially heterogeneous, and often unstable interface [59]. Also, hydrogen bubbles may form and hinder electrolytes from being exposed to the anodic surface, reducing the effective electrochemically active area [30]. This evolving surface chemistry, together with the transport of hydroxide (OH^−^) and aluminate (Al(OH)_4_^−^) ions between the electrode interface and the bulk electrolyte, establishes a delicate and transient balance between passivation, de‐passivation, and localized corrosion (Figure 2) [60]. As a consequence, corrosion frequently manifests in non‐uniform patterns, most notably pitting at microstructural defects and inclusions, leading to accelerated material degradation [19]. These processes collectively result in uncontrolled hydrogen gas evolution, progressive consumption of active Al, voltage instability under load, and limited anode utilization, all of which compromise the overall efficiency and cycle life of the battery [61]. By integrating surface engineering, inhibitor chemistry, and electrolyte design, it becomes possible to significantly mitigate corrosion and enable durable, high‐performance metal–air battery systems [62, 63, 64, 65, 66]. Together, these strategies highlight the complex interplay between interfacial chemistry, electrochemical kinetics, and additive‐driven surface modification necessary to stabilize Al anodes [56, 67, 68].

Additives in the electrolytes serve to precisely modulate surface reactions, interfacial chemistry, and reaction kinetics, operating through multiple synergistic mechanisms that collectively improve battery efficiency, durability, and power output [69, 70]. By targeting both the anode and the cathode, as well as the bulk solution, additives in electrolyte provide a multi‐level approach to controlling parasitic processes and optimizing electrochemical performance [71]. The corrosion inhibition behavior of some organic, inorganic, and hybrid additives (ZnO, Na_2_SO_4_, acetic acid, thiobenzamide, ethylenediaminetetraacetic acid (EDTA), glucose, etc.) is studied through a comprehensive theoretical framework combining density functional theory (DFT), and molecular dynamics simulations (MDS) methods [56, 72, 73, 74, 75, 76]. These complementary computational techniques provide both electronic‐level and dynamic deep insights into the adsorption and protective mechanisms operating at the Al metal–electrolyte interface. Thus, by integrating quantum chemical descriptors with interfacial simulations, a robust structure–activity relationship between additive molecular architecture and inhibition efficiency is established.

From a quantum chemical perspective, DFT calculations were employed to evaluate key electronic parameters governing molecular reactivity and adsorption propensity. The energies of the frontier molecular orbitals, HOMO and LUMO, play a pivotal role in describing donor–acceptor interactions between additive molecules and the Al surface. A higher HOMO value enhances the electron‐donating capability of the additive, facilitating charge transfer to vacant orbitals of surface Al atoms, whereas a lower LUMO value increases the molecule's ability to accept back‐donated electrons from the metal. Consequently, a reduced HOMO–LUMO energy gap signifies increased molecular polarizability and reactivity, promoting stronger adsorption and more effective surface coverage [56, 74, 76]. This enhanced interaction stabilizes the adsorbed layer and suppresses anodic dissolution and cathodic hydrogen evolution reactions.

Additional global reactivity descriptors, such as absolute hardness (*η*) and softness (*σ*) values, further support this interpretation. Lower *η* and higher *σ* values indicate that the additive molecules can readily undergo electronic rearrangement during adsorption, strengthening interfacial bonding [72, 74]. The electrophilicity index and electronegativity provide further insight into the balance between electron acceptance and donation, which governs adsorption stability. Importantly, the calculated fraction of electrons transferred (ΔN) from the additive to the Al surface is positive for effective additives, confirming spontaneous electron donation and chemisorption‐driven interactions. Such charge transfer processes are essential for the formation of a compact and adherent protective film on the Al metal surface, as demonstrated for Beta (+) D‐glucose and Adonite additives in NaOH (4 m) electrolyte for Al alloy anode whose ΔN values of 5.098 and 5.967 were calculated, respectively [72]. To capture the dynamic nature of inhibitor adsorption, MDS simulations may be also conducted to examine the time‐dependent behavior and preferred configurations of additive molecules at the Al–electrolyte interface [72]. As an example N‐α‐Fmoc‐N‐epsilon‐Boc‐D‐lysine (FDLH), amino acid, additive in alkaline Al–air cell, predominantly adopt a nearly parallel orientation relative to the Al surface [56]. This configuration maximizes the contact area between the molecule and the substrate, enabling multiple adsorption centers such as heteroatoms, π‐electron systems, and functional groups to interact simultaneously with surface Al atoms [56]. This multi‐point attachment mechanism significantly enhances adsorption strength and film stability. Moreover, the high adsorption energies obtained from MDS confirm the thermodynamic favorability of additive binding, which is widely recognized as a key indicator of effective corrosion inhibition [72]. The results showed that organic additives such as thiobenzamide (TBA) molecules preferentially adsorb onto the Al surface with more negative adsorption energies than those of water molecules [72]. This preferential adsorption leads to the formation of a continuous protective layer that effectively blocks the access of H_2_O and OH^−^ to the metal surface, thereby mitigating corrosion processes [72].

In the case of inorganic–organic hybrid inhibitor systems, in‐depth experimental XRD results and DFT confirmed the deposition of a Zn protective film on the Al surface in alkaline media containing ZnO and organic acid (such as acetic acid, citric acid, and EDTA) additives [76]. To clarify the synergistic inhibition mechanism, adsorption simulations DFT of organic acids on the Zn surface were performed. The results demonstrated that organic additives can also adsorb strongly onto the Zn surface through chemical bonding, contributing to the stabilization of the Zn film. This dual adsorption behavior highlights a synergistic protection mechanism: the organic acid directly inhibits Al corrosion through adsorption on Al, while simultaneously protecting the Zn layer from oxidation and dissolution. As a result, the integrity and longevity of the Zn protective film are significantly enhanced, leading to superior overall corrosion resistance.

**FIGURE 3:**
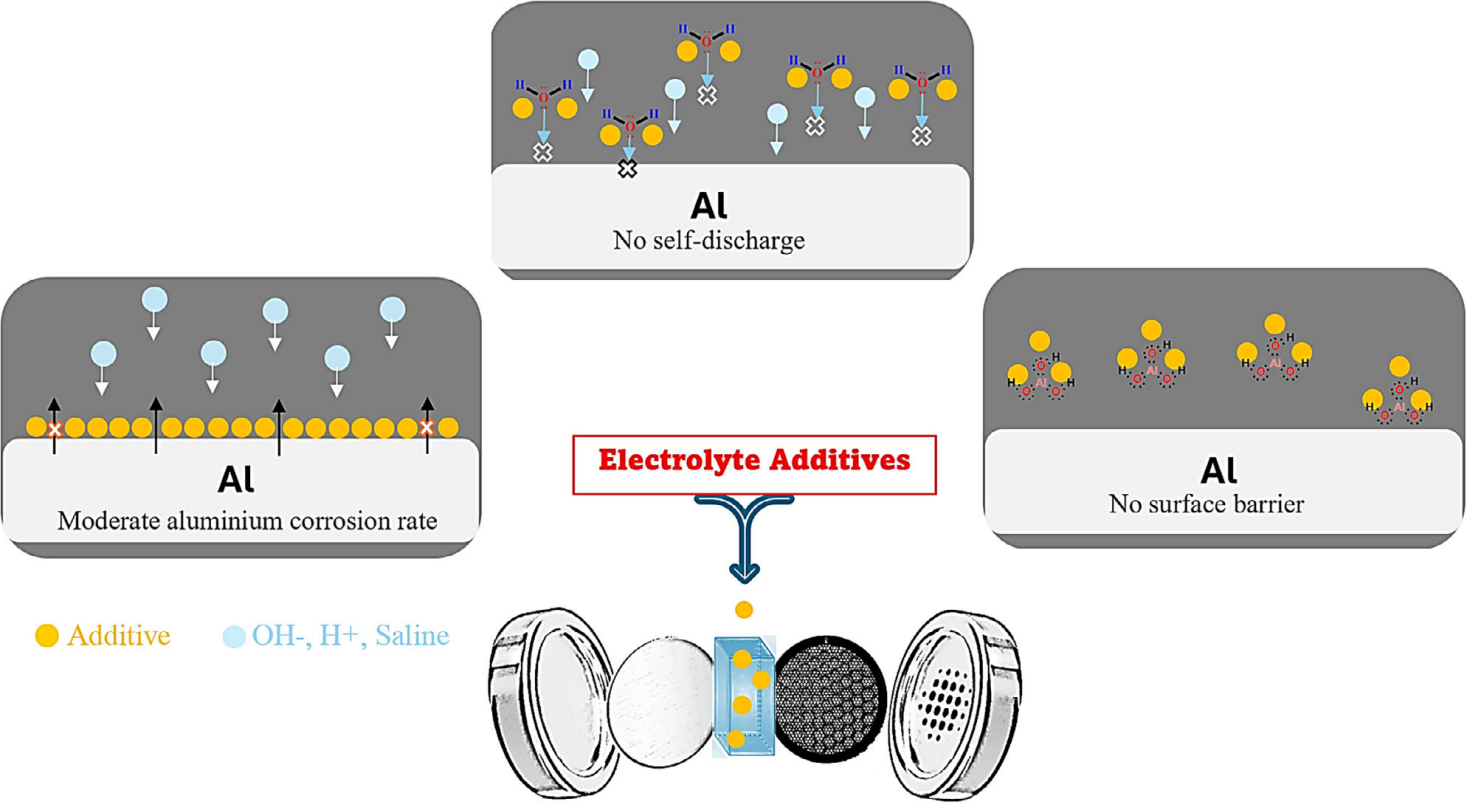
Scheme of additive mechanisms in metal–air battery.

By correlating experimental observations with theoretical predictions, distinct inhibition mechanisms of additives are proposed for single‐component additives (organic or inorganic) and for inorganic–organic hybrid systems such as 2‐mercaptobenzothiazole (MBT) / ZnO, and organic acids / ZnO [74, 76]. Basically, in controlling of corrosion, they follow three action mechanisms, namely (1) forming a protective film (2) catalytic effect (3) complexing agent (Figure 3) [77, 78, 79, 80, 81].

1. Corrosion inhibitors may form stable, protective films on the metal anode surface [64]. These films can develop through physical adsorption, chemisorption, or a combination thereof, effectively blocking active anodic sites, where metal atoms dissolve, and cathodic sites, where water reduction generates hydrogen gas [82]. Organic inhibitors, often containing heteroatoms such as nitrogen, oxygen, or sulfur, provide strong binding to the metal surface, while inorganic inhibitors may form insoluble complexes or precipitates that adhere firmly to the surface [73]. By limiting direct contact between the metal and aggressive electrolyte species, such as OH^−^, Cl^−^, or other anions, these inhibitors significantly reduce self‐corrosion, hydrogen evolution, and localized attack, including pitting and crevice formation [77].
2. Catalytic modifiers additives, such as ZnCl_2_ primarily target the air cathode, where the oxygen reduction reaction (ORR) occurs [83]. By interacting with catalysts such as doped metal oxides, these additives reduce ORR overpotentials, accelerate electron transfer, and increase reaction current density. Enhanced ORR not only improves battery power output but also stabilizes the electrochemical environment, indirectly mitigating anodic corrosion by maintaining balanced electron flow and preventing local overpotential spikes that would otherwise accelerate hydrogen evolution or passive layer breakdown.
3. Complexing and scavenging agents provide additional protection by regulating the solution chemistry of the electrolyte [80]. These additives bind free metal ions, such as Al^3^^+^, Mg^2^^+^, or Zn^2^^+^, forming stable complexes that prevent uncontrolled precipitation of hydroxides or oxides, which could otherwise obstruct pores, reduce ionic conductivity, and induce heterogeneous corrosion [84]. Scavenging agents additionally capture dissolved oxygen radicals or other reactive species capable of degrading the passive layer or the electrolyte itself, further stabilizing the system. These agents slow the dissolution of the native oxide layer and minimize localized alkalization, which is a major contributor to pitting and non‐uniform corrosion [84].

The optimal amount of additive in a metal–air battery electrolyte is determined by multiple interdependent factors, including the chemical nature of the additive, the composition of the electrolyte, the type and surface area of the metal anode, the operating conditions, such as temperature and current density, the desired balance between corrosion inhibition, catalytic enhancement, and ionic conductivity. For corrosion inhibitors, both organic and inorganic, typical effective concentrations range from the millimolar (mm) scale to low weight percent (0.01–1 wt%), depending on solubility and adsorption characteristics [64, 73, 85]. Organic inhibitors, such as agar, gelatin, or heteroatom‐containing molecules, often require higher concentrations to ensure sufficient surface coverage, as adsorption may be partial or reversible, whereas inorganic inhibitors, including Zn^2^^+^, or Sn^2^^+^, cations, can be effective at lower concentrations due to their ability to form insoluble protective films or complexes with Al^3^^+^ at the interface [76, 77, 86, 87, 88]. Additive efficacy further depends on molecular binding strength: strongly adsorbing molecules achieve effective protection at lower concentrations, whereas weakly adsorbing species may require higher doses or continuous replenishment.

In the following paragraphs, after a brief overview of the key metrics used to quantify corrosion and its mitigation through electrolyte additives, we will discuss the main additive categories, inorganic, organic, and hybrid organic–inorganic, also elucidating their impact on the electrochemical performance of Al–air batteries.

### 2.1 Key Metrics to Assess Anodic Corrosion in Al–Air Batteries

To gather comprehensive details on corrosion analysis, researchers take into account of some key metrics to assess corrosion in Al–air batteries and in particular, corrosion rate, inhibition efficiency, as well as anode utilization efficiency. We will briefly discuss the procedure of these methods in the following.

#### 2.1.1 Corrosion Rate

The corrosion rate or self‐corrosion assessment is found through Al weight loss as well as hydrogen evolution and electrochemical tests.

##### 2.1.1.1 Weight Loss

A specific size of Al is cut, washed with acetone and distilled water, air‐dried, and kept over a desiccant. The initial weight (W1) is measured and then utilized in an Al–air battery for a designated duration. The oxidized Al is taken from the battery cell, rinsed with distilled water and dried, then submerged in diluted nitric acid (HNO_3_) solution for 2–3 min to eliminate the corrosion residues. Ultimately, the oxidized Al was rinsed with distilled water, dried, and subsequently weighed again to determine the final weight (W2). Weight loss is indicated by the calculation shown below: [89]

ΔW=W1−W2

Another way to clean the Al surface is by using a solution containing CrO_3_ with H_3_PO_4_ in deionized water at about 90°C [90]. Standard procedures for assessing corrosion rate by Al weight loss using the following equation: [91, 92]

$$CR=\frac{k\cdot \Delta W}{A\cdot t\cdot \rho }$$

where *k* is a constant (8.76 × 10^4^), *ΔW* is the weight loss g, *t* is time h, *A* is Al surface area cm^2^, and *ρ* is Al density gcm^−3^ so that *CR* is in millimetre per year according to the standard guide for laboratory immersion corrosion testing of metals, ASTM G31‐12a, 2012. The electrolyte with drastic additives will have a lower corrosion rate.

##### 2.1.1.2 Hydrogen Evolution

A simple setup is used for hydrogen collection. The Al specimen is put into the electrolyte in a flask, and produced hydrogen is measured volumetrically by directing it into a burette. The number of gram moles is calculated from the hydrogen volume by the gas laws, and according to the self‐corrosion reaction the hydrogen moles number is 3/2 to the number of dissolved Al moles. This method also permits the measurement of the Al weight‐loss rate. This setup can be modified by connecting to a potentiostat to achieve specified results while the hydrogen evolution is measured [93].

##### 2.1.1.3 Electrochemical Tests

These tests are very efficient and offer a straightforward method for assessing the corrosion behavior of Al anodes. Various polarization techniques, such as cyclic voltammetry, potentiodynamic polarization, and potentiostaircase, are commonly employed for laboratory corrosion research. The potentiodynamic polarization analysis, often refer to as the Tafel test, is the most widely used polarization testing method for assessing corrosion. In this experiment, the Al–air battery is constructed using an Al anode within regulated test conditions. The potentiodynamic polarization measurements were conducted using a standard three‐electrode system, the counter electrode is Pt sheet, the working electrode is Al, and the reference electrode is Hg/HgO or Ag/AgCl. The open circuit potential (OCP) is measured in an electrolyte containing an additive and without an additive in 60 mins before the electrochemical test. Tafel polarization curves are recorded from −0.5 to 1.5 V versus OCP at a rate of 1 mV.

Standard guidelines to study corrosion rate by the below formula (ASTM G59–97, 2020):

$$\mathrm{CR}=3.27\times 10^{-3}{i\;}_{\mathrm{corr}}\cdot \frac{E_{w}}{\rho }$$

Which *i_corr_* is corrosion current density from the Tafel plot, *E_w_* is the equivalent weight of Al g, *ρ* is density of Al gcm^−3^ so that *CR* is in millimetre per year.

#### 2.1.2 Inhibition Efficiency

The efficiency of additive inhibition is typically assessed through polarization resistance measurements and also the hydrogen evolution test. The corrosion current densities (*i_corr_*) in the Tafel plot are extrapolated both with and without the additive, relying on the equation below:


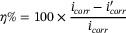

where *i*_corr_ is the corrosion current density without additive and *i'*_corr_ is the corrosion current density with additive.

In electrochemical impedance spectroscopy (EIS) analysis, the effectiveness of the additive in inhibiting is determined by the charge‐transfer resistance *R_ct_* without the additive and *R'_ct_* with the additive, according to the following equation: [94]

$$\eta \%=100\times \frac{R_{\mathrm{ct}}-{R^{\prime }}_{\mathrm{ct}}}{R_{\mathrm{ct}}}$$

Besides, the inhibition efficiency is calculated through hydrogen evolution by the below equation [76]:

$$\eta \%=100\times \left(1-\frac{R_{add}}{R_{0}}\right)$$

where *R*_0_ is the hydrogen evolution rate without additive and *R*_add_ is hydrogen evolution rate with additive. The hydrogen evolution rate is measured according to the following equation [95]:

$$R=\frac{V_{H2}}{A\cdot t}$$

where hydrogen evolution rate unit is mL cm^−2^ min^−1^, *A* is Al area cm^2^, *V* is the hydrogen collected volume mL, and *t* is the time min.

#### 2.1.3 Al Anode Utilization Efficiency

The anode utilization efficiency indicates the fraction of Al anode material that is assumed to provide current. The discharge test is studied in varied current densities in fixed discharge current in certain time. The energy density, capacity density, and anode utilization are calculated by the following equations: [96]

$$Aluminum\;utilization\%=100\times \frac{It}{\left(\frac{\Delta wF}{9}\right)}$$

where the time *t* is in s; the weight loss *Δw* is in g; anode utilization is in %; and *F* is the Faraday constant. The performance of the Al anode in Al–air battery is investigated by capacity density and energy density with additive in electrolyte and without additive.

$$Capacity\;density=\frac{Ih}{\Delta w}$$

$$Energy\;density=\frac{UIh}{\Delta w}$$

where the current *I* is in A; the time *h* is in h; the weight loss *Δw* is in g; the average voltage *U* is in V.

### 2.2 Organic Additives

In recent years, synthetic corrosion inhibitors have become one of the most effective methods of anode corrosion prevention in metal–air batteries. It has been observed that organic inhibitors adsorb onto the metal surface via their active parts, which reduce active sites on the metal surface, thereby decreasing the metal dissolution and providing inhibitory effects [97]. This adsorption of inhibitors via functional groups such as non‐paired electrons of hetero atoms or unsaturated bonds in aromatic units, which results in electronic interaction with the metal d orbitals [98]. It is noteworthy to mention that the surface chemisorption or physisorption of inhibitors increases with increasing their molecular mass, dipole moment, and the concentration of the inhibitor [99]. The orientations of organic inhibitors on the metal surface are regulated by pH values and electrode potentials [97]. These factors can influence the adsorption behavior and effectiveness of the inhibitors.

**FIGURE 4:**
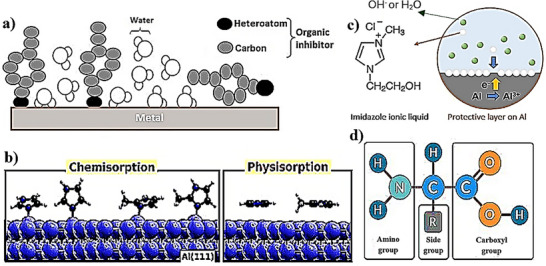
(a) Organic inhibitors adsorption onto the metal surface. (b) Chemisorption and physisorption of imidazole on Al surface [100]. (c) Protective layer of imidazole ionic liquid on Al [101]. (d) Basic structure of amino acids.

Organic inhibitors containing nitrogen, sulfur, and oxygen heteroatoms or double or triple bonds are known as highly efficient inhibitors for Al–air batteries (Figure 4a) [35, 102]. Their structure typically includes an aromatic heterocyclic moiety and functional groups such as ─NO_2_, ─NH_2_, ─OH, ─COOR, ─COOH, or ─CONH_2_. In the following, we will present the recent developments in organic additives on Al–air batteries, such as ionic liquids, surfactants, amino acids, carboxylic acids, hydrogen‐bonds, and other organic additives as corrosion inhibitors. It has to be mentioned here that additives always refer to alkaline electrolytes.

#### 2.2.1 Ionic Liquids

Ionic liquids are molten salts, i.e. ionic salts having a low melting point below room temperature. The inhibition efficiency of ionic liquids is dependent on the nature and length of the alkyl chain, kind of anion, the concentration of ionic liquid in the solvent, and temperature [103]. Ionic liquids used as additives in Al–air batteries comprise tetra‐alkylated ammonium, and *N,N′* ‐dialkylated imidazolium salts with various alkyl groups, with anions being either an organic or inorganic anion as presented in Table 3.

**TABLE 3:** Ionic liquid additives in Al–air batteries.

	Ion liquid additive	Ion liquid additive structure
*N, N*‐dialkylated imidazolium	1‐hexadecyl‐3‐methylimidazolium hexafluorophosphate [103]	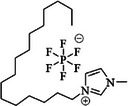
	1‐allyl‐3‐methylimidazolium bis(trifluoromethylsulfonyl)imide [104]	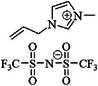
	1‐(2‐hydroxyethyl)‐3‐methylimidazolium chloride [101]	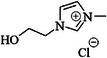
tetra‐alkylated ammonium	1‐butyl‐3‐methylimidazoliumhexafluorophosphate [105]	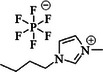
	tris(2‐hydroxyethyl) methyl ammonium methylsulfate [106]	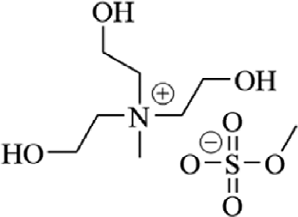

*N,N’*‐dialkylated imidazole is a five‐membered heterocyclic organic compound containing 2 nitrogen atoms with delocalized electrons and an aromatic ring structure. *N,N’* ‐dialkylated imidazole‐based ionic liquids have higher inhibition efficiency than other ionic liquids [101]. Primarily, this is due to its superiorities, such as the positive charge on the nitrogen atom, which is distributed through resonance within the aromatic ring (a polarizable cation) [107]. Second, the aromatic ring π‐electrons can interact with the metal d‐orbitals, which prevents metal dissolution. Thirdly, the unpaired electrons on the nitrogen atoms, which can adsorb onto metal surface thereby enhancing the inhibition efficiency. It is important to note that the adsorption of the imidazole on the Al surface can occur in either planar orientation or upright position with the nitrogen atom oriented toward the metal surface. This orientation is influenced by pH and electrode potentials (Figure 4b) [100, 108].

The formation of a protective layer with imidazolium ionic liquid in alkaline media occurring at the anode is shown in Figure 4c. It is generally accepted that absorbed water molecules on a metal surface can be replaced with organic inhibitors. The corrosion inhibition mechanism of imidazolium‐based ionic liquid additives is started by the replacement shown in Equation (1) at the anode, which forms a monolayer on the Al surface [109]. This monolayer polarity on the Al surface induces the adsorption of another imidazolium ionic liquid layer on the adsorbed complex, resulting in the formation of multilayer complex Equation (2).

(1) $$\begin{array}{c} {\left[\mathrm{IM}\right]}^{+}X^{-}+H_{2}O\left[\mathrm{Al}\;\mathrm{surface}\right]\to \left[IM^{+}\;\mathrm{Al}\;\mathrm{surface}\right]X^{-}+H_{2}O\\ X^{-}=Cl^{-},\;OH^{-} \end{array}$$

(2) $$\begin{array}{c} {\left[\mathrm{IM}\right]}^{+}X^{-}+\left[IM^{+}\;\mathrm{Al}\;\mathrm{surface}\right]X^{-}\to X^{-}{\left[\mathrm{IM}\right]}^{+}X^{-}\left[IM^{+}\;\mathrm{Al}\;\mathrm{surface}\right]\\ X^{-}=Cl^{-},\;OH^{-} \end{array}$$

The dipole moment interaction is the main reason to generate stable multilayers on the Al surface. Besides, there are long alkyl chains driving a more packed layer by Van der Waals forces. The alkyl groups may be oriented horizontally or vertically on the surface of Al, which can form aggregation and cause a reduction in corrosion rate and restrict the Al dissolution [110]. Obviously, the distribution of charge, polarizability, and electronegativity of anionic moieties of organic inhibitors effect on the inhibition efficiency [111].

It is noteworthy that the maximum efficiency of ionic liquid additives in water is achieved at a certain concentration, which is attributed to the ionic liquids' self‐aggregation (is named as critical aggregation concentrations). This aggregation is affected by the length of the alkyl chain, the nature of counter anions, and their interactions with water [112]. The overview of reports on ionic liquid organic additives in Al–air battery is presented in Table 4.

**TABLE 4:** Overview of reports on ionic liquid organic additives in Al–air battery.

Additive (%)	Anode	Cathode	Electrolyte	Al's utilization (%)	Specific capacity density (mAhg^−1^) (discharge current)	Energy density (Whkg^−1^)	Inhibitory efficiency (%)
1‐(2‐hydroxy ethyl)‐3‐methyl imidazolium chloride (7 mm) [101]	Al‐5052	Bi_2_O_3_ based catalyst	NaOH (4 m)	41.2 (blank), 82.9 (with additive)	1227 (blank), 1269 (with additive) (20 mAcm^−2^)	1595 (blank), 3313 (with additive)	58.2
1‐ hexadecyl‐3‐ methyl imidazolium hexafluoro phosphat*e* (4 mm) [103]	Al‐5052	MnO_2_	NaOH (4 m)	34.7 (blank), 71.4 (with additive)	1034 (blank), 2128 (with additive) (20 mAcm^−2^)	1220 (blank), 2554 (with additive)	58.4
1‐allyl‐3‐methylimidazolium bis(trifluoromethylsulfonyl)imide (1.5 mm) [104]	Al	Not mentioned	NaOH (4 m)	51.4 (blank), 93.8 (with additive)	1720 (blank), 2554 (with additive) (20 mAcm^−2^)	—	96.2
1‐butyl‐3‐methyl imidazolium hexafluoro phosphate (0.8 gL^−1^) [105]	Al‐6061	Not mentioned	KOH (4 m)/ ethylene glycol (30%)	24.2 (blank), 66.2 (with additive)	719 (blank), 1971 (with additive) (20 mAcm^−2^)	767 (blank), 1668 (with additive)	71.9
tris(2‐hydroxyethyl) methyl ammonium methylsulfate (200 ppm) [106]	Al	MnO_2_/C	KOH (4 m)	60.2 (blank), 93.8 (with additive)	1793 (blank), 2795 (with additive) (20 mAcm^−2^)	—	66.0

#### 2.2.2 Surfactants

Another important category of organic additives is surfactants (surface‐active agents), which feature hydrophobic and hydrophilic moieties, rendering them amphipathic (*amphis* = both and *philia* = friendship in Greek) [113]. Normally, they are used to reduce surface tension (e.g. to improve substrate wettability). In the Al–air systems containing surfactants, the Gibbs free energy is lower by surfactant molecules aggregation at the electrolyte and electrode interface rather than remaining as individual molecules in the bulk solution. Surfactants can adsorb on solid surfaces, a property which can lead to affecting corrosion efficiency. The surface‐active agents are categorized based on the charge of their head to four groups including 1‐ non‐ionic (uncharged), 2‐ anionic (negatively charged), 3‐ cationic (positively charged), and 4‐ amphoteric (carries both a positive and a negative charge) (Table 5) [114].

**TABLE 5:** The surfactant additives in Al–air batteries.

Category	Name	Structure
Non‐ionic	Nonoxynol‐9 [115]	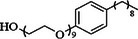
	n‐octyl phosphonic acid [116]	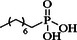
Cationic	Dodecyl dimethyl benzyl ammonium bromide [117]	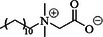
Amphoteric	Betaine [118]	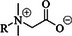
Hybrid	Dodecyl‐β‐D‐maltoside & Dodecyldimethyl benzyl ammonium bromide [119]	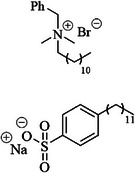

The mechanism by which surfactant additives inhibit self‐corrosion of the Al surface involves the formation of a protective layer on the surface. The double electric layer functioning as a protective layer consists of a combination of hydrophilic and hydrophobic molecules. Besides, an electrostatic interaction exists between the positive center and the negative center in the surfactant structure in alkaline media. Surfactants interact and adsorb on the hydrophilic surfaces of Al by their hydrophilic moiety part. As for high alkane chains (16 C>long chain>12 C) have the favorable hydrophobicity, so the compact protective layer on Al surface enhances corrosion efficiency [120]. An overview of reports on surfactant additives in Al–air battery is presented in Table 6.

**TABLE 6:** Overview of reports on surfactant additives in Al–air battery.

Additive (%)	Anode	Cathode	Electrolyte	Al's utilization (%)	Specific capacity density (mAhg^−1^) (discharge current)	Energy density (Whkg^−1^)	Inhibitory efficiency (%)
Nonoxynol‐9 (2 mm) [115]	Al	MnO_2_/C	NaOH (4 m)	15 (blank), 25 (with additive)	1772 (blank), 2320 (with additive) (20 mA cm^−2^)	—	92.8
n‐octyl phosphonic acid [116]	Al	MnO_2_/C	NaOH (4 m)	53 (blank), 79 (with additive)	1579 (blank), 2353 (with additive) (20 mA cm^−2^)	—	63.5
Dodecyl dimethyl benzyl ammonium bromide (0.6 mm) [117]	Al	MnO_2_/C	NaOH (4 m)	—	1240 (blank), 2430 (with additive) (20 mA cm^−2^)	—	56.3
Betaine (1 mm) [118]	Al	Not mentioned	NaOH (4 m)	53.1 (blank), 66.6 (with additive)	1580 (blank), 1980 (with additive) (25 mA cm^−2^)	396.3 (blank), 2365.1 (with additive)	81.0
Dodecyl‐β‐D‐maltoside (1 mm) & Dodecyldimethyl benzyl ammonium bromide (0.8 mm)/ 8‐hydroxyquinoline (10 mm) [119]	Al‐5052	Mixed of Pt/C, tetra methoxy porphyrin cobalt, tetra methoxy porphyrin iron, and Mn_x_O_y_/Ag	NaOH (4 m)	84.5 (with additive)	2517 (with additive) (20 mA cm^−2^)	3297 (with additive)	87.2

#### 2.2.3 Amino Acids

Amino acids are organic compounds containing an amino [─NH_2_] and carboxyl [─COOH] functional group as well as a side group R. Amino acids have different properties based on their R group (Figure 4d) [121]. One of the most important candidates of amino acids is heterocyclic amino acids, which exhibit an aromatic ring as the side group that possesses at least one atom other than carbon, such as nitrogen or sulfur. It has been presented that the heterocyclic amino acids and their derivatives, due to donor−acceptor interaction with the metal surface can form a protective film on it [122, 123, 124, 125]. Consequently, amino acid derivatives can act as corrosion inhibitors in metal air batteries. There are some reports on using histidine and tryptophan, which contain an imidazole and indole side group, respectively, as alkaline corrosion inhibitors for Al−air batteries. Lei Guo et al. introduced eco‐friendly tryptophan amino acid derivatives, *L*‐tryptophan, (*tert*‐butoxy carbonyl)‐*L*‐tryptophan and *N^α^‐*(*tert*‐butoxy carbonyl)‐*N1*‐formyl‐*L*‐tryptophan as the corrosion inhibitors for Al–air battery. In‐depth analyses of its inhibitory effect and influence on the performance of the Al−air batteries were conducted [122, 124, 125].

It is shown that following the addition of *L*‐tryptophan (90 mm) as an inhibitor additive in NaOH (4 m) electrolyte for Al–air battery, the discharge potential increased and remained steady [122]. The anode utilization value increased three times more than the blank solution, which has reached 90.76%. The energy density increased from 1317.23 to 4094.59 Whkg^−1^ (considering the Al weight) and the capacity density increased from 990.1 mAhg^−1^ (blank) to 2702.7 mAhg^−1^ (with additive). The outcome demonstrated that the discharge performance of the battery inhibitor‐containing was stable, and the overall voltage increased after adding a corrosion inhibitor (discharge current: 20 mA cm^−2^). By replacing one hydrogen in ─NH_2_ group with *tert*‐butoxy carbonyl in *L*‐tryptophan structure the performance of Al−air battery is interestingly changed, the addition of just 2 mm (*tert*‐butoxy carbonyl)‐*L*‐tryptophan in NaOH (4 m) electrolyte (45 times less than *L*‐tryptophan) significantly increased the Al anode capacity density to 2739.7 mAhg^−1^, the anode utilization value increased 91.9% and energy density 3723.53 Whkg^−1^ (considering the Al weight) [125]. The enhancement could be attributed to the role of the groups of the organic ligands, which play an important role in the inhibitor molecule coordination bonding with the Al surface, reducing the active sites, inhibiting hydrogen evolution, and improving the discharge voltage while lowering self‐corrosion. By replacing a hydrogen in indole with formyl group in (*tert*‐butoxy carbonyl)‐*L*‐tryptophan structure, it has exhibited lower performance of Al−air battery compared to the *L*‐tryptophan [125]. It is worth to mention that the addition of the *N^α^*‐(*tert*‐butoxy carbonyl)‐*N1*‐formyl‐*L*‐tryptophan (1.5 mm) as inhibitor additive in NaOH (4 m) electrolyte improves the overall voltage, the capacity and energy density and the anode utilization efficiency to 2469.1 mAhg^−1^, 3384.6 Whkg^−1^, and 82.9%, respectively, compared to blank electrolyte. According to this study, *L*‐tryptophan derivatives have shown good corrosion‐inhibiting effects on the Al anode in an alkaline environment, following the order (*tert*‐butoxy carbonyl)‐*L*‐tryptophan > *L*‐tryptophan > *N^α^*‐(*tert*‐butoxy carbonyl)‐*N1*‐formyl‐*L*‐tryptophan > blank.

Histidine is one of the most important heterocyclic amino acids, which features an imidazole R group in its structure. Histidine is applied as a corrosion inhibitor for Al–air batteries [123]. It is shown that by the addition of histidine (50 mm) in KOH (4 m) electrolyte in Al–air battery, the current density of Al electrode increased to 40 mAcm^−2^, compared with the blank. The corrosion inhibition efficiency and corrosion current density values were 43.3% and 7.5 mAcm^−2^, as for the blank 0% and 13.2 mAcm^−2^, respectively.

#### 2.2.4 Carboxylic Acids

**FIGURE 5:**
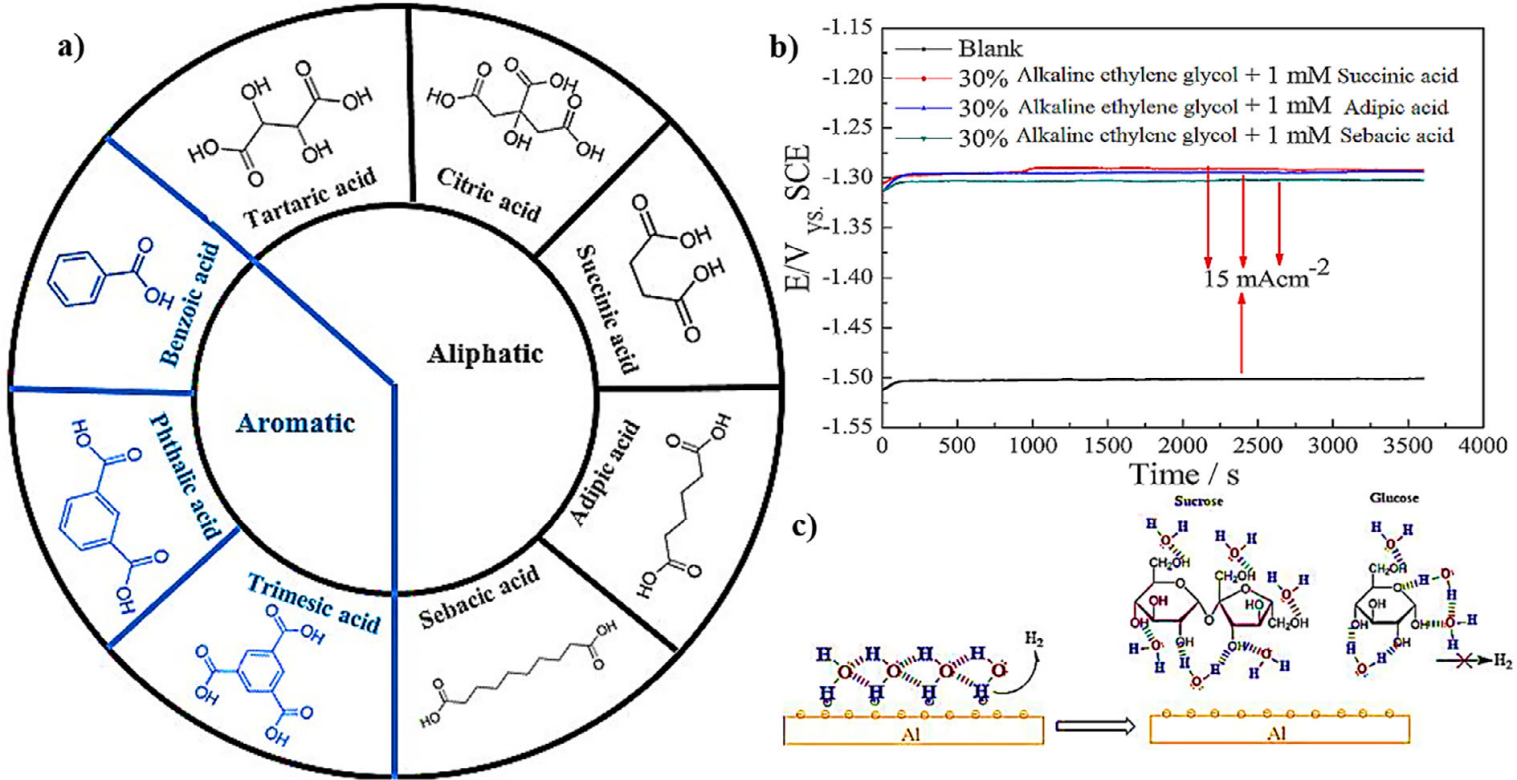
(a) Aliphatic and aromatic carboxylic acids additives. (b) The discharge curve of aliphatic and aromatic carboxylic acids additives [127]. (c) The sucrose and glucose trap water molecules and break in the inert hydrogen bonds network.

The carboxylic acid functional group, ─COOH, is one of the major polar functional groups in organic additives. It can slow down the Al anode corrosion in alkaline media due to coordination with Al atoms on the anode surface [126]. The carboxylic acid additives can be divided into two categories, including aliphatic and aromatic (Figure 5a). The main difference between aliphatic and aromatic carboxylic acids is that the carboxylic acid functional group is attached to an aliphatic carbon chain, which mostly has a linear structure in its *R* group, whereas in aromatic carboxylic acids, an aromatic ring is cyclic and features resonance structures.

It is important to note that the effectiveness of aromatic acids as inhibitors is influenced by the number and position of the carboxylic acid functional groups and other substituents on the aromatic ring. Also, the chain length of the aliphatic carboxylic acid functional group plays an important role in inhibiting power, by increasing the chain length the inhibiting efficiency is increased [128, 129]. Brito et al. investigated the influence of aliphatic carboxylic acids on the performance of Al in alkaline media [126]. In this study, the additives were divided into three groups according to their functional groups, including aliphatic carboxylic acids, amino acids, and quaternary amines. The aliphatic carboxylic acid, such as *L* (+) tartaric acid (10.0 gL^−1^) with 35% efficiency and citric acid (10.0 gL^−1^) with 38% efficiency, are proved as efficient inhibitors in NaOH (6 m) electrolyte for the Al corrosion reaction, the efficiency increased with increasing the number of acid groups in the compound. As a result of this study, the inhibition efficiency of the three groups is in the following order:

quaternaryamines>aminoacids>aliphaticcarboxylicacids

Zhang et al. have used aliphatic dicarboxylic acids such as succinic acid, adipic acid, and sebacic acid as electrolyte additives in the alkaline ethylene glycol electrolyte for commercial AA5052 Al alloy anode in Al–air batteries [127]. The average discharge voltage is reported to be −1.291, −1.295, −1.302 V in the applied electrolyte with succinic acid, adipic acid, and sebacic acid, respectively. Also, the Al anode capacity density increased to 2773, 2811, and 2777 mAhg^−1^, the anode utilization values were 91.1%, 93.2%, 94.5%, and 93.3% in the NaOH (4 m) electrolyte for blank, containing succinic acid, adipic acid, and sebacic acid, respectively. The discharge curve showed a good discharge plateau in low potential for AA5052 alloy anode due to the stable complex of Al^3+^ cations and dicarboxylic acids on the anode surface (Figure 5b). We have to mention that the potential of the blank cell is −1.5 V with an anode efficiency of 91% at the current density of 15 mAcm^−2^. Despite a maximum improvement of 3% in anode utilization, this research found a 0.3 V decreasing of the potentials using this additive!

Wang et al. studied the performance of three aromatic carboxylic acids namely benzoic acid, iso‐phthalic acid, and trimesic acid as corrosion inhibitors on Al‐7075 anode for Al–air battery in KOH (2 m)/ethanol (volume ratio 8/2) electrolyte [130]. The results showed that all of the three aromatic acids are effective inhibitors for Al–air battery with the following inhibition efficiency order: benzoic acid > iso‐phthalic acid > trimesic acid. The average discharge voltage with the addition of aromatic acids declined and stabilized at 0.924, 0.912, and 0.909 V in alkaline electrolyte containing benzoic acid, iso‐phthalic acid, and trimesic acid, respectively. On the other hand, the capacity density significantly improved from 1017.81 mAhg^−1^ to 1357.47, 1291.14, and 1199.15 mAhg^−1^ and anode utilization rose from 32.9% to 45.6%, 43.4%, and 40.3% in alkaline electrolyte containing benzoic acid, iso‐phthalic acid, and trimesic acid, respectively.

#### 2.2.5 Hydrogen Bonds

Alkaline aqueous media contain large amounts of H and O atoms, in which oxygen is highly electronegative. Thus, hydrogen bonds, O─H⋅⋅⋅O, are created between H atoms and O atoms, and the result is a H‐bond network between water molecules, which allows for an ultra‐fast diffusion of ions. If water (H_2_O) is easily transferred to the electrode surface, the subsequent unwanted redox reactions can easily occur and yield in hydrogen production. Therefore, disrupting the H‐bond network in alkaline media will contribute to a decrease of the hydrogen production reaction. Recent reports have shown that hydrogen bond rich organic additives effectively break the H‐bond networks in alkaline aqueous media (Figure 5c). Tang et al. utilized sucrose and glucose as hydrogen bond‐rich organic additives to inhibit the corrosion for Al–air batteries [131, 132].

The donor‐acceptor interaction of vacant Al d‐orbitals with free electrons of additives on the metal surface atoms is the main reason for the adsorption of glucose and sucrose on the Al anode surface [131]. Sucrose and glucose with abundant hydroxyl groups can trap water molecules and break the inter H‐bond network of water molecules, thereby reducing the water molecule's reactivity to avoid hydrogen production reaction. Consequently, the Al self‐corrosion rate has significantly decreased, and the Al–air battery performance is considerably increased. The elements of Al alloy (wt%) in this study are Mg 0.024, Ga 0.011, Sn 0.010, Zn 0.004, Fe ≤0.009, Cu ≤0.001, Si ≤0.001, Al remainder, and the commercial Mn_x_O_y_@Ag catalyst film attached to different sides of the Cu mesh is used as cathode. The Al alloy self‐corrosion rate reduced from 1.3 to 0.17 mgcm^−2^min^−1^, inhibition efficiency of 87%, and 2330 mAhg^−1^ specific capacity is reported for sucrose‐containing electrolyte. Besides, the Al discharge time extended from 6 hg^−1^ to 22 hg^−1^, proving high practical application. On the other hand, in 3 m of glucose additive in NaOH (4 m) the specific capacity increased to 2886.7 mAhg^−1^, energy density to 3675.1 Whkg^−1^, and the inhibition efficiency was up to 98% at 5 mAcm^−2^. Also, the free water fraction decreased to 48.7% from 88.0%, which is calculated with reactive force field and MDS. This demonstrates that the introduction of glucose and sucrose as hydrogen bond‐rich additives successfully decreased the free water molecules on Al surface. It has been demonstrated that the addition of organic solvents in aqueous electrolytes with high ability of hydrogen bonding, such as dimethyl sulfoxide and glycerol is also an effective method to impact the H‐bond network, thus helping to suppress side reactions on the electrode in Al–air batteries [133, 134]. The lowest hydrogen evolution rate of 0.02 and 0.042 mLmin^−1^cm^−2^ was found for dimethyl sulfoxide and glycerol, respectively. Besides, the Al–air battery containing dimethyl sulfoxide presented the highest energy density of 3106.04 Whkg^−1^ and a discharge capacity of 2271 mAhg^−1^ at 0.05 mA cm^−2^, and energy density of 2102.6 Whkg^−1^ and a discharge capacity of 2564 mAhg^−1^ for glycerol‐containing electrolyte.

#### 2.2.6 Other Organic Additives

Research focusing on the development of environmentally friendly, non‐toxic, biodegradable, low bioaccumulation, and no harmful elements materials as corrosion inhibitors has also found recent interest. For example, Naderi et al. have used plant extracts from extracted from Mentha piperita L/Lawsonia inermis, Flax Straw, and Chrysanthemum Coronarium leaves, respectively to improve the Al–air batteries performance. An overview of other additives is summarized in Table 7.

**TABLE 7:** The overview of reports on plant extract additives in Al–air battery.

Additive (%)	Anode	Cathode	Electrolyte	Al's utilization (%)	Specific capacity density (mAhg^−1^) (discharge current)	Energy density (Whkg^−1^)	Inhibitory efficiency (%)
Mentha piperita L (800 ppm)and Lawsonia inermis (800 ppm) [135]	Al‐5083	Pt/C	NaOH (2 m) /ethylene glycol, the volume ratio of 7:3.	92.1 (blank), 94.1 and 93.6 (with additive)	2743 (blank), 2805 and 2789 (with additive) (10 mA cm^−2^)	—	88.7 and 87.9
Flax Straw (3 vol %) [136]	Al	MnO_2_/C	KOH (5 m)	—	63 mAhcm^−2^ (25 mA cm^−2^)	—	62.0
Chrysanthemum coronarium leaves (4.5 g L^−1^) [137]	Al	a commercial gas diffusion electrode	NaOH (4 m)	—	920.25 (blank), 2941.18 (with additive) (10 mA cm^−2^)	938.65 (blank), 2970.59 (with additive)	11.6 (blank), 95.1 (with additive)

Moreover, organic compounds containing hetero atoms such as nitrogen, oxygen, and sulfur have also shown to be excellent inhibitors of Al corrosion. This includes urea, thiourea, 6‐thioguanine, and thiobenzamide [73, 138, 139]. Generally, inhibitor containing S, O, and N atoms together have better inhibition efficiency. A better performance seems to be presented in the order: O < N < S. Thiobenzamide has both N and S atoms in its conjugated structure and it is encouraging that the inhibition efficiency of thiobenzamide goes up to 67.8%, with the specific capacity greatly enhanced from 461.9 mAhg^−1^ (blank) to 1532.6 mAhg^−1^ in KOH (5 m).

The utilization of inorganic additives represents one of the most cost‐effective strategies to mitigate self‐corrosion in Al–air batteries. These additives are typically incorporated into the electrolyte or electrode materials [140, 141]. Three typical categories can be defined: zinc‐based inorganic additives, sodium‐based inorganic additives, and miscellaneous inorganic additives. It is well‐established that the adsorption of cations on Al and oxyhydroxides can occur below the point of zero charge, the pH value at which the net electrical charge on a solid material's surface is zero, which is influenced by the solution composition and metal phase composition [142]. There is empirical evidence suggesting that anions like chloride, sulfate, acetate, and carbonate, when adsorbed on Al surfaces, induce additional negative charges, thereby promoting the adsorption of cations [143].

### 2.3 Inorganic Additives

In a recent study, zinc‐based compounds (e.g. ZnO, ZnCl_2_, ZnCO_3_) were demonstrated to be promising additives [144]. A protective layer, consisting of zinc (Zn) and its oxide or hydroxide, effectively restrains self‐corrosion by mitigating the hydrogen evolution while having minimal impact on the anodic reaction of Al [144]. This Zn coating effectively mitigates the water reduction reaction, leading to a reduced OH^−^ ions concentration and consequently inhibiting the formation of corrosion products [144]. Due to these difficulties in the formation of Al corrosion products, the Al^3+^ cations generated from anodic reaction predominantly migrate into the bulk solution [144]. Consequently, the discharge performance of Al–air batteries has been markedly improved. In addition, the mitigation of the water reduction reaction on Al anodes facilitates the efficient transfer of electrons generated from anodic dissolution to the air cathode under operating conditions. This enhances the power output during discharge and significantly improves the coulombic efficiency of Al–air batteries [144]. The energy distribution of Al electrode is shown in Equation (3).

(3) $$E_{\mathrm{Al}}=E_{c}+E_{i}+E_{h}$$

The *E_Al_* is the total energy of Al; *E_c_* is the energy consumed by Al discharge; *E_i_* is the energy consumed by Al reaction with Zn additive, and *E_h_* is the energy consumed by Al self‐corrosion. The utilization rate of the Al electrode can be improved by reducing the *E_h_* as much as possible.

The Al is soaked in blank electrolyte NaOH (4 m), and with Zn additive for 1 h, then the XRD analysis has used to elucidate the mechanism of the corrosion inhibitor to prove that the corrosion products deposited on the Al surface [145]. Equation (4) shows the Al anode self‐corrosion in alkaline electrolyte. According to Equations (5) and (6), it can be deduced that Zn initially forms from Zn(OH)^2−^_4_ on the Al surface, which is then oxidized to Zn oxide by the oxygen present in water, see Equation (7). In addition to the diffraction peaks attributed to Al, peaks for Zn and ZnO are also observed, suggesting that they are the primary components of the deposited protective layer.

(4) $$2\mathrm{Al}+6H_{2}O+2OH^{-}\to 2\mathrm{Al}{{\left(\mathrm{OH}\right)}^{-}}_{4}+3H_{2}$$

(5)

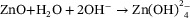



(6)

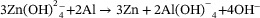

(7) $$2\;\mathrm{Zn}+O_{2}\to 2\mathrm{ZnO}$$

When zinc hydroxide is deposited on an Al anode, zinc hydroxide nuclei are initially generated on the surface. The zinc hydroxide ion is concentrated in these first nucleation sites, called hot spots, and this major factor leads to non‐homogeneous layer of zinc hydroxide on the Al surface. Therefore, it is possible to solve this problem by constructing a structure that can maintain the transport of zinc hydroxide homogeneously. For example, a 3D stainless steel mesh is applied on the Al anode surface to encapsulate the anode, which provides a relatively large surface area to provide higher homogeneity of hot spots on Al surface, so the anode efficiency reported is higher than without mesh. Besides, the ZnO layer film is detached, and the ZnO is accumulated gradually until it falls off. The mesh is installed on the outer surface of the anode and has a gap with the anode surface. Also, the mesh should not be a part in the electrochemical reaction, but it has good conductivity, low cost, and easy maintenance, which is why stainless‐steel is a good solution.

Wei et al. recently developed a high‐performance Al–air battery with a mesh‐encapsulated anode and a ZnO saturated in KOH (6 m) electrolyte [17]. This design enabled efficient transfer, recovery, and utilization of energy from the Zn film on the Al anode surface, achieving a specific capacity of 1839.8 mAhg^−1^ and an energy density increase of 102%. However, the Zn film was loose and unstable, resulting in suboptimal anode and cell capacity efficiency. To address this issue, a strong inorganic Lewis acid, ZnCl_2_, was used as an additive in the alkaline electrolyte, leading to an enhanced‐performance Al–air battery. This study suggests that the protective effect on the Al anode is further enhanced by the adsorption of anionic groups formed by Cl^−^ in the alkaline electrolyte, which improved energy utilization during discharge, resulting in superior output performance, with a specific capacity of 2322 mAhg^−1^ and an anode efficiency of 77%. The effect of Zn ion percent saturation in the electrolyte on Al self‐corrosion remains poorly understood. Furthermore, a combination was created where Zn(OH)_4_^2−^ and OH^−^ coexist. Zn forms a deposit on the surface of the Al electrode, effectively mitigating self‐corrosion. The maximum inhibition efficiency, approximately 73.9%, is observed when the Al alloy (composition: Al 99.6, Fe 0.35, Si 0.25, Zn 0.05, Cu 0.05, Ti 0.03, Mg 0.03, Mn 0.03 wt%) sheet is immersed in a 6 M KOH electrolyte containing ZnO. The results demonstrate that the incorporation of ZnO enhances discharge performance, and the capacity is further increased using a mesh‐encapsulated anode, where the mesh size is 1.5 × 1.5 mm^2^ (06Cr19Ni10) is installed on the outer surface of the Al alloy and has a gap on it. Among these, the cell featuring a mesh‐encapsulated anode reveals an anode efficiency of approximately 61%. The capacity is approximately 1839 mAhg^−1^, while the specific energy is about 2099 mWhg^−1^. These values correspond to increases of 101% and 102%, respectively, compared to without mesh.

Since aluminum hydroxide (Al(OH)_3_) has a low solubility in water, the dissolution will increase in high‐concentration acid and base, it can affect the anode efficiency when Al(OH)_3_ accumulates on the surface. The electrolyte is responsible for the transport of Al and OH^−^ from the anode surface. Pumps are used in the traditional Al–air flow batteries to flow the electrolyte, which requires a complicated water management system [146]. Nowadays, microfluidic Al–air batteries often adopt porous paper or cotton cloth as the flow channel to deliver the electrolyte [147, 148]. Yang et al. have fabricated a microfluidic Al–air battery with ZnO as the inhibitor [149]. The ZnO 0.5 gL^−1^ in 3.5 m KOH solution electrolyte, integrated with a microfluidic structure, achieves a peak power density about 18 mWcm^−^^2^, an open circuit voltage greater than 1.6 V, and a specific capacity of 2335 mAhg^−1^ at a current density of 20 mAcm^−^^2^, while exhibiting a low corrosion rate of 0.3 mLcm^−^^2^min^−1^ hydrogen evolution rate. This performance duration is 1.18 times longer than microfluidic batteries without ZnO additive and 6 times greater than non‐microfluidic batteries. This pattern demonstrates a corrosion inhibition effect of the ZnO additive. The observed corrosion inhibition is due to ZnO ability to lower the corrosion potential of the Al anode, −1.36 V and −1.77 V in KOH 7 m [150, 151].

Zinc additives, in addition to being effectively used in encapsulated anode with mesh and also flow batteries, have shown positive effect on classic Al–air batteries too. Pino et al. have demonstrated the impact of alloying elements and the incorporation of ZnO and ZnCl_2_ as corrosion inhibitors in the *N,N′*‐methylene‐bisacrylamide / acrylic acid alkaline gel electrolyte [152]. ZnCl_2_ was found to be less effective as a corrosion inhibitor compared to ZnO for the Al7475 clad alloy at a constant discharge current density of 0.8 mAcm^−^^2^. A galvanic couple is generated between Al and Zn when the electrolyte contains an additive contacted with the Al alloy. The process resulted in the formation of a protective layer that mitigated Al corrosion and enhanced the potential for hydrogen generation.

Wei et al. also employed ZnCl_2_, ZnO, and ZnCO_3_ as additives in KOH (6 m) electrolytes, thereby emphasizing the superior performance of ZnCl_2_ [153]. With an increase in Zn^2^^+^ concentration, the passivation effect on the anode surface is progressively alleviated, resulting in the formation of a more stable and compact Zn film. Additionally, the corrosion current decreases progressively, reflecting a gradual reduction in hydrogen evolution at the anode. Finally, the effectiveness of various additives in inhibiting anodic self‐corrosion at the same concentration can be ranked as follows: ZnCl_2_> ZnCO_3_> ZnO. When 0.5 m ZnCl_2_ is employed as an additive, the battery attains the best specific capacity of approximately 2322.9 mAhg^−1^ at 20 mAcm^−2^ and a specific energy of 2457 WhKg^−1^, with an anode efficiency of up to 77.9%.

Hosseini et al. have utilized a range of sulfur‐oxide‐containing compounds, namely K_2_S_2_O_8_, Na_2_SO_4_, Na_2_SO_3_, C_6_H_5_SO_2_OH, and C_2_H_6_SO as additives to evaluate their effects on self‐corrosion and hydrogen evolution reactions at the Al anode electrode [75]. The maximum observed inhibition efficiency was approximately 43% at 10 mAcm^−2^, achieved using an electrolyte of 10 vv^−1^% C_6_H_5_SO_2_OH/KOH (4 m). The inhibition efficiencies of the additives were ranked as follows because of SO groups presence and the formation of Al_2_(SO_4_)_3_ soluble salt in the alkaline aqueous solution: C_6_H_5_SO_2_OH > C_2_H_6_SO > Na_2_SO_4_> Na_2_SO_3_> K_2_S_2_O_8_> KOH. The cathodic branches of the polarization curves for electrolytes containing K_2_S_2_O_8_, Na_2_SO_4_, Na_2_SO_3_, C_6_H_5_SO_2_OH, and C_2_H_6_SO are less than KOH (4 m) (without additive). The presence of additives moderately influences the anodic branch of the polarization curves, with no significant changes detected in the anodic dissolution of the Al electrode. It has been reported that the K_2_S_2_O_8_ additive reduced discharge performance relative to blank KOH (4 m) electrolyte, which is attributed to the sulfate radicals (SO_4_^−^) intermediate. On the other hand, the discharge capacities increased by 29%, 18%, 1.3%, and 16% with the incorporation of Na_2_SO_4_, Na_2_SO_3_, C_6_H_5_SO_2_OH, and C_2_H_6_SO into the KOH (4 m) electrolyte, respectively, compared to the without additives. The Al–air battery incorporating Na_2_SO_4_ as the best additive achieves a specific capacity of 2604 mAhg^−^^1^ (based on the mass of consumed Al), surpassing the 2021 mAhg^−^^1^ capacity observed with the additive‐free electrolyte. From polarization and power curves, the Na_2_SO_4_/KOH electrolyte achieved a maximum power density of 94 mWcm^−^^2^ at a current density of 217 mAcm^−^^2^, significantly outperforming the 60 mWcm^−^^2^ observed with a 4 m KOH solution at a current density of 150 mAcm^−^^2^.

**FIGURE 6:**
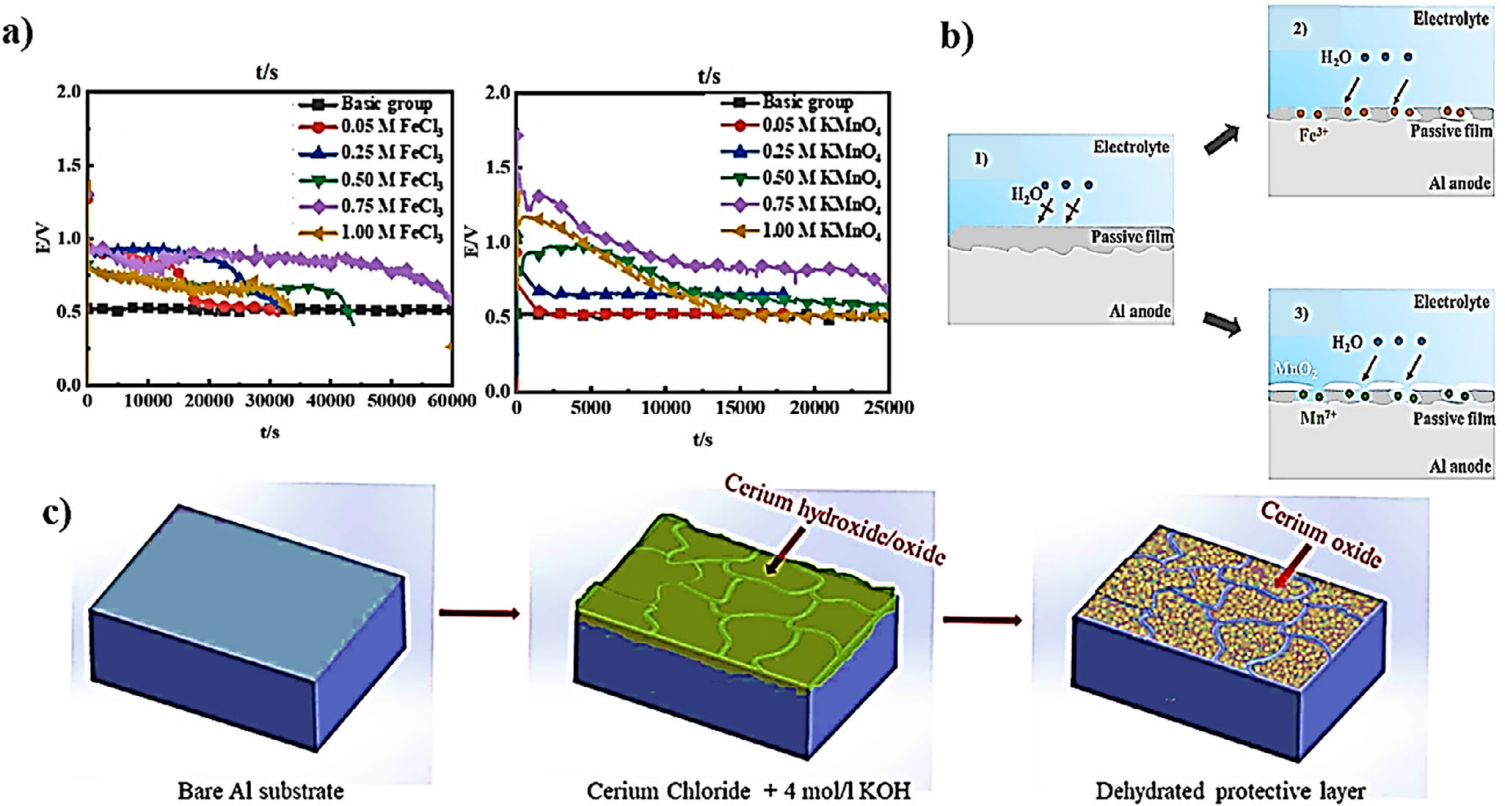
(a) The long‐term discharge testing FeCl_3_ and KMnO_4_, (b) The passive film formation on the Al anode with FeCl_3_ and KMnO_4_ [154]. (c) absorption mechanism of cerium on the Al surface [155].

Inorganic oxidants such as FeCl_3_ and KMnO_4_ have been introduced as additives in 3.5% NaCl solution electrolyte for Al–air battery [154]. The discharge voltage remarkably increased from 0.56 V for blank electrolyte to 1.18 and 1.15 V (at constant current discharge) by adding FeCl_3_ and KMnO_4_, respectively (Figure 6a). It has been mentioned that the oxidant additives can activate Al atoms on anode surface due to the reaction between Al and Fe^3+^ or MnO_4_^−^, which the battery discharge time is increased. The passive film ruptured and continuous dissolution of Al anode during the discharge process. MnO_2_ was accumulated on the surface by adding KMnO_4_. Consequently, the oxidation of Mn^2+^ occurred on the surface, as shown in Figure 6b.

Cerium is the most abundant of the rare earths, and it has been shown to also inhibit the redox reactions on Al anodes. Thus, it is beneficial to add an inhibitor for Al alloys [156]. Cerium chloride, is introduced as a self‐corrosion inhibitor for Al–air batteries in KOH (4 m) electrolyte [155]. It is reported that by increasing the cerium chloride concentration, the capacity density increased from 1294 mAhg^−1^ for blank to 2244 mAhg^−1^ with the additive at 20 mAcm^−2^, and the anodic efficiency increased from 43.8% for blank to 76.1% with the additive. At higher concentrations of cerium chloride, the Al anode surface was saturated when the concentration of cerium chloride reached 1.5 wt%, indicating the uneven deposition of the cerium hydroxide precipitates on the anode surface, which restricted a further increase in inhibition efficiency. On the other hand, by increasing the cerium chloride concentration, a cerium hydroxide layer formed on Al surface and the discharge voltage moved from −1.97 V to more positive values −1.85 V. Interestingly, by increasing the immersion time from 30 min to 60 min in KOH 4 M before adding cerium chloride to show its healing behavior before carrying out the EIS analysis, the corrosion resistance increased by over 300%. It is reported that the Ce^3+^ can create a stable passive layer with hydroxide on the Al surface (Figure 6c). An overview of reports on metal‐based additives in Al–air battery is summarized in Table 8.

**TABLE 8:** The overview of reports on metal‐based additives in Al–air battery.

Additive (%)	Anode	Cathode	Electrolyte	Al's utilization (%)	Specific capacity density (mAhg^−1^) (discharge current)	Energy density (Whkg^−1^)	Inhibitory efficiency (%)
ZnO (0.35 g) [152]	Al7475	Carbon & MnO_2_ coated on Ni mesh, double‐cathode cell design	acrylic acid‐ N,N‐ methylene‐bisacrylamide gel in KOH (16 mL, 11 m)	—	256 (with additive) (0.8 mA cm^−2^)	—	—
ZnO saturated [17]	Al1060 encapsulated in 06Cr19Ni10 mesh	Carbon coated on foamed nickel	KOH (6 m)	—	1484.3 (blank), 1839.4 (with additive) (20 mA cm^−2^)	1498.8 (blank), 2099.1 (with additive)	49.8 (blank), 61.7 (with additive)
ZnO (0.5 gL^−1^) [149]	Al6061	Not mentioned	KOH (3.5 m)	37	1090 (with additive) (10 mA cm^−2^)	1700 (with additive)	45.3
ZnCl_2_ (0.5 m) [153]	AA1060	MnO_2_ and carbon coated on foamed nickel	KOH (6 m)	—	2322 (with additive) (20 mA cm^−2^)	2457 (with additive)	77.9
Na_2_SO_4_ (10% v/v, 1 m) [75]	Pure Al (>99%)	MOF (Co_3_O_4_/NC‐CNT)	KOH (4 m)	67 (blank), 87 (with additive)	2021 (blank), 2604 (with additive)	60 mW cm ^−2^ (blank), 94 mWcm ^−2^ (with additive) (217 mA cm^−2^)	50.0
CeCl_3_.7H_2_O (1%) [155]	Pure Al (>99%)	Graphite	KOH (4 m)	67.8%	1294 (blank), 2244 (with additive) (20 mA cm^−2^)	—	43.8 (blank), 76.1 (with additive)

### 2.4 Organic–Inorganic Hybrid Additives

Although organic inhibitors gained much attention for their low toxicity and ease of decomposition [104, 127, 157, 158, 159]. Compared with inorganic additives (such as ZnO, ZnCl_2_, or CaO), their corrosion inhibition effect is limited by their relatively low conductivity in high pH solution [160, 161]. At the same time, in terms of inorganic additives, their corrosion inhibition efficiency interferes with their solubility in electrolytes and results in uneven and loose deposition of the protecting coating [162]. To push the limit of single additives, research has been devoted to exploring diverse combinations of organic/inorganic additives.

One of the most crucial functions of organic additives in hybrid additive systems is constructing a stable linking bridge between the inorganic inhibitors and Al electrodes. The adsorptive force of the polar functional groups in organic additives works on the Al electrode and the inorganic additives simultaneously, converting the loose protective inorganic film to a tense hybrid layer on the Al electrode [163]. For example, as mentioned above, adding the Zn‐contained corrosion inhibitor could hinder hydrogen evolution due to Zn's higher hydrogen evolution over‐potential than Al. Nevertheless, the loose and spongy Zn layer would gradually detach because of the evolution of H_2_, causing the exposure of the Al substrate to the alkaline electrolyte [95].

**FIGURE 7:**
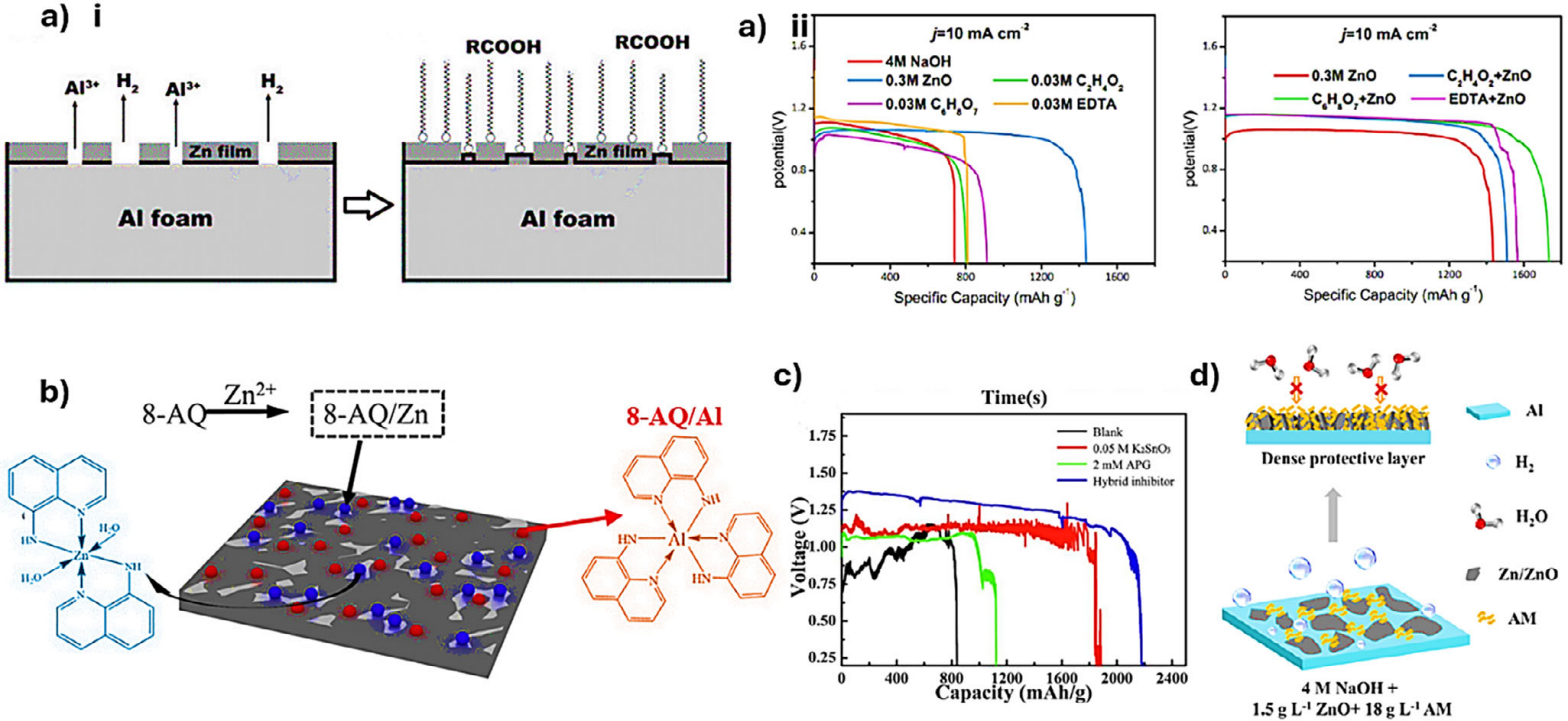
(a‐i) The protective films of ‐COOH and ZnO on Al surface, (ii) Discharge curves of single inhibitor and hybrid inhibitors [76]. (b) 8‐AQ coordinated bond with Al^3+^ and Zn^2+^ and chemical structure of H_2_QS on Al surface [164]. (c) Discharge curves of single K_2_SnO_3_, APG inhibitors, and hybrid inhibitor [165]. (d) The formation of a sheet‐like film of inorganic on the Al surface in AM presence [145].

Utilizing the strong adsorption of an organic inhibitor on the Al anode and its diverse bond system formed with Zn^2+^, the distribution of the Zn protective layer can become uniform and dense. Jiang et al. [76] further compared the corrosion inhibition effect and the corresponding mechanism of three hybrid additives: ZnO (3 m) hybrid with citric acid (C_6_H_8_O_7_) (0.03 m), or EDTA (0.03 m), or acetic acid (C_2_H_4_O_2_) (0.03 m) in NaOH (4 m). The inhibition efficiency of the hybrid of ZnO + C_6_H_8_O_7_ reached 86.9%, compared to that of single ZnO, which only reached 59% at 0.3 m. This significant synergistic effect is caused, on the one hand, by the organic acid inhibiting the oxidation of Zn film, and on the other hand, as shown in Figure 7a‐i, by protective films that are further strengthened by the adsorptive force between ‐COOH groups in organic acid, ZnO (RCOO‐Zn), and Al surface (RCOO‐Al). The discharge curves of the Al–air battery with single and ZnO/organic acid hybrid additives are shown in Figure 7a‐ii. As anticipated, the ZnO/organic acid hybrid additives significantly enhanced the discharge performance and improved anode corrosion resistance.

The existence of organic additives can also impact the inorganic additive's deposition. Dithiothreitol (DTT) can adsorb on the surface of Al with the formation of a six‐membered ring complex by the most active site ‐SH groups [166]. Wu et al. utilized this active site as the connector between the Al surface and Zn^2+^ ions, which lowered the adsorption of Zn^2+^ on the electrode surface, leading to an even Zn deposition layer [167].

Next, modified quinolines can also form linker bonds between the Al electrode and inorganic additives. Li et al. [164] made use of 8‐aminoquinoline (8‐AQ) as a bi‐dentate ligand, which formed a covalent bond and coordinated bond with Al^3+^ and Zn^2+^, stabilizing the Zn layer on the surface of Al (Figure 7b). With the cooperation between 1.0 mm 8‐AQ and 1.6 gL^−1^ ZnO electrode's inhibitor, the maximum corrosion inhibitive efficiency and electrode's utilization with NaOH 4 m solution as the electrolyte are as high as 83.3% and 90.8% (47.0% for blank). Similarly, by the introduction of the sulfonic group to the quinoline ring, 8‐hydroxyquinoline‐5‐sulfonic acid (H_2_QS) is a bidentate chelating agent with two electrophilic substituents, namely, it has a strong electrostatic interaction with inorganic additives and the Al electrode at the same time. With surface morphology analysis and the adsorptive state simulation, Zhang et al. constructed a complete and compact film formed by ring structures with 4 H_2_QS units linked by 2 Ca^2+^ ions. The maximum corrosion inhibition efficiency rose from 46.8% (CaO), 51.6% (H_2_QS) to 86.3% (CaO + H_2_QS) with 4 m NaOH solution using as the electrolyte [168].

Another inorganic protective coating resulting from stannate solutions can also be strengthened by the addition of organic additives (Equation (8)). Wang et al. utilized the guiding effect of alkyl polyglucoside (APG additive 1) on the reduction of potassium stannate (additive 2) in an alkaline electrolyte, promoting the even deposition of Sn film on the Al anode, thus putting down the hydrogen evolution effectively [165].

(8)

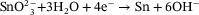




Owing to its hydroxyl groups and long carbon chain, APG can not only adsorb on the metal surface, hindering hydrogen evolution with the isolation effect, but also regulate the deposition of Sn film, causing a uniform Sn‐protecting layer on the anode. Compared with the single inhibitor, the inhibitor efficiencies increase from 80.6% (2 mm APG), 86.9% (0.05 m K_2_SnO_3_) to 94.1% (2 mm APG + 0.05 m K_2_SnO_3_), the discharge curves of single inhibitors and hybrid inhibitor are shown in Figure 7c).

Moreover, casein, ethylene glycol, potassium sodium tartrate, ellagic acid (EA), and hydroxyethyl cellulose, with the strong adsorption of their polar functional groups, all play a synergistic role in electrolyte systems by cooperating with SnO_3_^2−^ [169, 170, 171, 172, 173]. Furthermore, sodium alginate, hydroxyethyl cellulose, carboxymethyl cellulose, poly(ethylene glycol) di‐acid, triethanolamine are all also reported for the reinforcement of the inorganic protective layer [144, 173, 174, 175, 176].

Reversely, the inorganic additive can also promote the adsorption of organic inhibitors. Hu et al. proved the adsorption energy between ethylene glycol (EG) and the Al surface with the help of Sn in Na_2_SnO_3_ is significantly higher than without [170]. At the same time, EG enables a uniform deposition of tin and improves the protective effect of Na_2_SnO_3_ on the Al electrode. The electrolyte with simultaneous presence of 0.05 m Na_2_SnO_3_ and 15 wt% EG resulted in corrosion inhibition efficiencies as high as 34.1%, corresponding to 12.5% and 27.3% when they were used alone in 4 m NaOH electrolyte.

Tiron (4,5‐dihydroxybenzene‐1,3‐disulfonic acid) has the deprotonated form, which is a strong complexing agent and can form an Al‐Tiron complex, preventing the Al ions from reacting further with OH^−^ ions. Palanisamy et al. discovered NaNO_3_, not only minimizing the H_2_‐evolving corrosion rate, but also facilitating the deprotonation of Tiron. The mixed electrolyte (0.005 m Tiron + 0.005 m NaNO_3_ can significantly enhance the kinetics of the Al electrode in 0.5% NaCl electrolyte [79].

In the past decade, the concept of “water‐in‐salt” electrolyte has attracted the attention of many researchers working on aqueous batteries. In such a concentrated solution, the interionic interaction becomes dominant over the solvent–ion interaction, resulting in reducing the water availability and a broad electrochemical window [177, 178]. Tang et al. introduced a water‐in‐salt electrolyte, potassium acetate–KOH, for Al–air batteries, which clearly increased the inhibition corrosion efficiency of the Al anode and enhanced the discharge capacity. Yet, the utilization of this water‐in‐salt electrolyte had low ionic conductivity and high viscosity so impeding their practical applications. Therefore, they regulated the electrolyte formulation to overcome the above issues and reduce the free water molecules to achieve the high‐performance Al–air batteries [179]. By introducing sodium stannate, Na_2_SnO_3_ (0.02 m), as a highly efficient additive in high‐concentration potassium acetate‐KOH electrolyte (8, 4 m, respectively), the corrosion potential of the Al alloy anode is significantly increased from −1.39 to −1.53 V in 8 m potassium acetate (KOAc) in 4 m KOH electrolyte as the hybrid high concentration electrolyte (Al alloy composition: Mg 0.024%, Ga 0.011%, Sn 0.010%, Zn 0.004%, Fe ≤0.009%, Cu ≤0.001%, Si ≤0.001%, Al remainder). After the presence of sodium stannate the activity of water molecules was reduced, a protective layer was formed on the anode surface, the decomposition of water molecules on the surface and corrosion reaction of the Al anode were successfully lowered, and the highest inhibition efficiency reached 87.6%. The mass specific capacity of the Al–air battery containing Na_2_SnO_3_ in high‐concentration electrolyte (water‐in‐salt) and blank high‐concentration electrolyte were 2439 mAhg^−1^ and 600 mAhg^−1^, respectively.

Hosseini et al. have investigated organic and inorganic additives with acetate functional groups as corrosion inhibitors for Al–air battery. Inhibitor efficiencies of calcium acetate hydrate, barium acetate hydrate, copper acetate hydrate, iron acetate hydrate, nickel acetate hydrate, cobalt acetate hydrate, and ethyl acetate were studied [82]. The corrosion current densities are reported in the order of Ba‐acetate < Ca‐acetate < ethyl‐acetate < blank, while the other metal salts have not shown good result. An Al–air battery containing Ba‐acetate additives demonstrated a higher specific capacity than Ca‐Acetate, ethyl‐Acetate, and blank ones. Furthermore, the energy density of Al–air batteries containing acetate additives has been reported to be 3014, 2378, and 2024 Whkg^−1^ for Ba‐Acetate > Ca‐Acetate > ethyl‐Acetate, respectively at 10 mAcm^−2^. The corrosion inhibition efficiency followed the order of: Ba‐Acetate > Ca‐Acetate > ethyl‐Acetate, with the highest inhibition efficiency of 50% for electrolyte containing Ba‐Acetate.

It was shown that there is an accumulation issue of reaction products in Al–air batteries, which is why research on introducing flocculants to overcome this drawback has been conducted. A recent study by Wang et al. has revealed that the addition of sodium polyacrylate as a flocculant additive in electrolyte can accelerate the sedimentation of discharge products without compromising the battery performance [180]. The carboxylate anion of polyacrylate coordinated with Al(OH)_3_ inhibits the accumulation on an Al surface. Besides, polyacrylate can form hydrogen bonds with water, thus reducing the free water molecules. Encouragingly, the mass‐specific capacity of Al was significantly increased to 1947 mAhg^−1^ from 909 mAhg^−1^ at 25 mAcm^−2^, and the highest inhibition efficiency reached 67.6% at 90% content of sodium polyacrylate.

Long‐chain polymers can also affect the crystal growth of the inorganic inhibitor and further cause the morphological change of the protective layer. Tian et al. found a strong blocking effect of polyacrylamide (PAM), which increases the competition between crystal growth and nucleation (Figure 7d) [145]. Without PAM, the rates of nucleation and growth are in equilibrium. However, the nucleation is prevented with PAM, leading to a decrease in the number of nuclei. Meanwhile, PAM could also adsorb the products to avoid crystals from directional growing on Al surface. These oriented crystals induce the formation of a sheet‐like film and wrap the film, which slows the dissolution of the inorganic layer and increases hydrophobicity between the anode and the electrolyte. An overview of reports on organic–inorganic hybrid additives in Al–air battery is summarized in Table 9.

**TABLE 9:** The overview of reports on organic–inorganic hybrid additives in Al–air battery.

Additive (%)	Anode	Cathode	Electrolyte	Al's utilization (%)	Specific capacity density (mAhg^−1^) (discharge current)	Energy density (Whkg^−1^)	Inhibitory efficiency (%)
10 mm 8‐hydroxyquinoline + 4.0 mm ZnO [95]	Al5052 alloy		NaOH (4 m)	56.2 (blank), 70.3 (with additive)	—	3267 (with additive)	40.3 (with additive)
0.3 m ZnO + 0.03 m citric acid [76]	3D porous Al foams	MnO_2_/C	NaOH (4 m)	58.2 (with additive)	763.2 (blank), 1902.0 (with additive) (10 mA cm^−2^)	740.3 (blank), 1735.4 (with additive)	24.8 (blank), 86.9 (with additive)
1.5 mm Dithiothreitol + 0.3 m ZnCl_2_ [167]	Al	Not mentioned	NaOH (4 m)	40.1 (blank), 60.2 (with additive)	1314.5 (blank), 1793.3 (with additive) (15 mA cm^−2^)	1340 (blank), 2047 (with additive)	0 (blank), 95 (with additive)
1.6 gL^−1^ ZnO + 1.0 mm 8‐aminoquinoline [164]	AA5052 alloy	Not mentioned	NaOH (4 m)	47.0 (blank), 90.8 (with additive)	1399 (blank), 2703 (with additive) (20 mA cm^−2^)	2043 (blank), 4068 (with additive)	74 (with additive)
0.08 gL^−1^ CaO + 0.5 mm 8‐hydroxyquinoline‐5‐sulfonic acid [168]	AA5052 alloy	Not mentioned	NaOH (4 m)	41.3 (blank), 90.7 (with additive)	1231 (blank), 2703 (with additive) (20 mA cm^−2^)	1806 (blank), 4002 (with additive)	86.5 (with additive)
0.05 m K_2_SnO_3_ + 2 mm alkyl polyglucoside [165]	Home‐made Al alloy	MnxOy@Ag	KOH (4 m)	28.6 (blank), 73.8 (with additive)	840 (blank), 2180 (with additive) (100 µA cm^−2^)	—	94.1 (with additive)
0.05 m Na_2_SnO_3_ + 0.6 gL^−1^ casein [169]	Home‐made Al alloy	MnO_2_	NaOH (4 m)	—	—	—	0 (blank), 75.1 (with additive)
15 wt.% EG + 0.05 m Na_2_SnO_3_ [170]	1060 Al sheets	Not mentioned	NaOH (4 m)	15.6 (blank), 43 (with additive)	—	543.3 (blank), 1577.9 (with additive)	24.4 (with additive)
SST (0.0005 m) + STT (0.0007 m) [171]	pure Al sheet	MnO_2_/C	KOH (1 m)	23.5 (blank), 69.2 (with additive)	35.3 mAhcm^−2^ (blank), 50 mAhcm^−2^ (with additive) (20 mA cm^−2^)	—	38.8 (with additive)
4 mm EA + 0.05 m Na_2_SnO_3_ [172]	Home‐made Al alloy	Mn_x_O_y_/Ag	KOH (4 m)	—	926 (blank), 2439 (with additive) (50 mA cm^−2^)	—	52.2 (with additive)
8 gL^−1^ ZnO + 10 gL^−1^ CMC [174]	AA5052 alloy	Not mentioned	NaOH (4 m)	91.1 (blank), 94.1 (with additive)	2710 (blank), 2824 (with additive) (15 mA cm^−2^)	—	70.3 (with additive)

In summary, hybrid organic–inorganic additives integrate the advantages of both additive types, creating a synergistic, multi‐level protection and performance‐enhancing system. At the Al anode, organic molecules adsorb through heteroatoms or π‐systems while inorganic ions or complexes simultaneously precipitate or react with surface hydroxides to form mixed Al–O–M passivating phases. The resulting hybrid films are mechanically stronger, chemically more stable, and capable of accommodating local pH fluctuations, effectively sealing defects, suppressing hydrogen evolution, and reducing parasitic corrosion. In the electrolyte bulk, hybrid additives stabilize dissolved Al^3^^+^ ions through combined organic complexation and inorganic complex formation, buffer local pH via coordinated acid–base equilibria, and lower water activity through polymer–salt or polyol–salt networks, preventing pore blockage in polymer electrolytes. At the air cathode, these systems enhance ORR kinetics by combining redox‐active organic mediators with catalytically active inorganic salts, lowering overpotential, improving power density, while scavenging reactive oxygen species to protect the carbon support and binders. Collectively, this coordinated action across the anode, electrolyte, and cathode results in superior anode utilization, extended cycle life, improved energy efficiency, enabling Al–air batteries with enhanced performance, durability, and operational stability compared to systems employing only organic or inorganic additives. For example the energy density increases from 1700 Whkg^−1^ by using only inorganic ZnO to 4068 Whkg^−1^using hybrid ZnO/8‐aminoquinoline additive [149, 164]. A concise summary of the mechanisms and effects of organic, inorganic, and hybrid additives in Al–air batteries components is presented in Table 10, highlighting their complementary strategies for corrosion mitigation and electrochemical enhancement.

**TABLE 10:** Comparison of organic, inorganic, and hybrid additives in Al–air batteries.

Additive Type	Primary Mechanisms	Action at Anode	Action in electrolyte	Action at cathode	Limitations / Notes
Organic	Adsorption, complexation, pH buffering	Forms flexible, conformal films that block anodic dissolution and cathodic hydrogen‐evolution sites	Complexes Al^3^^+^ ions, buffers local pH, reduces water activity, prevents Al(OH)_3_ precipitation	—	Stability is limited under high pH, high temperature, or long‐term cycling; excessive concentration can increase viscosity and reduce ionic conductivity
Inorganic	Rigid passivation, electrostatic stabilization, controlled precipitation, catalytic enhancement	Forms Al–O–M layers to block aggressive OH^−^ ions, increases hydrogen‐evolution overpotential	Stabilizes Al^3^^+^ speciation, prevents uncontrolled precipitation	Enhances ORR kinetics, stabilizes catalyst sites	Less adaptable to surface defects, limited dynamic adsorption, lacks redox mediation and complexation ability
Hybrid (Organic–inorganic)	Synergistic combination of adsorption, passivation, complexation, redox mediation, catalytic enhancement	Organic molecules adsorb and seal defects, inorganic ions form Al─O─M hybrid films; mechanically stronger and chemically stable	Organic ligands + inorganic complexes stabilize Al^3^^+^, buffer pH, reduce water activity; prevent pore blockage and film breakdown	Increase power density, stabilize catalysts	Provides multi‐level protection, highest anode utilization, extended cycle life, uniform anodic oxidation; combines advantages of both organic and inorganic additives

## 3 Future Outlook

Several challenges remain to be addressed to translate the beneficial effects of electrolyte additives from laboratory cells to practical Al–air devices. From a scaling perspective, many of the most effective additives reported so far have been optimized in small‐scale cells using highly purified electrolytes and carefully controlled conditions. Future studies should therefore systematically evaluate additive performance in larger‐format cells, under higher areal capacities, and with commercially relevant Al alloys and air cathodes. Particular attention should be paid to the cost and synthetic complexity of organic and hybrid additives and to their environmental and health footprint compared with simpler inorganic inhibitors. Only additives that remain effective at low concentration could be compatible with existing electrolyte preparation and cell manufacturing processes. If these will not introduce additional safety or environmental concerns, they will ultimately be viable for large‐scale deployment.

Long‐term stability represents another key bottleneck. Most reports demonstrate inhibition efficiency and performance enhancement over relatively short discharge times or a limited number of cycles, whereas practical applications especially for partially or fully rechargeable Al–air systems will require stable operation over hundreds to thousands of hours. Future work should therefore quantify not only initial corrosion rate suppression and anode utilization, but also the retention of these metrics under extended operation, intermittent cycling, and temperature fluctuations. This includes understanding how additives age, decompose or are consumed, how the composition and morphology of the protective interfacial films evolve with time, and how these changes impact hydrogen evolution, passivation, and the formation of solid by‐products. Coupling electrochemical testing with in situ/operando characterization and multiscale modelling will be essential to unravel degradation pathways and to design more robust additive systems.

Finally, there is significant potential for data‐driven and machine‐learning‐guided additive design. Inspired by recent advances in machine‐learning‐assisted optimization of catalysts and electrode materials in related metal–air systems [12], similar frameworks could be developed for Al–air electrolytes. High‐throughput quantum‐chemical calculations and molecular dynamics simulations, combined with experimental datasets of inhibition efficiency, corrosion rate, anode utilization and discharge performance, could be used to train predictive models that identify promising additive structures and multi‐component formulations. Active‐learning and Bayesian optimization strategies could then iteratively guide experiments toward the most informative candidates. In parallel, establishing standardized testing protocols and open databases for additive performance would greatly enhance the reliability of such approaches. Overall, integrating mechanistic understanding with data‐driven design is expected to accelerate the discovery of low‐cost, environmentally benign, and highly efficient additives, thereby bringing practical Al–air technologies closer to commercialization.

## Funding

Italian Ministry of University and Research PNC0000007, CUP B53C22006960001 and P20227BLHS, CUP B53D23025240001; Wallenberg Wood Science Center ID 63574; German Research Foundation: 390874152.

## Conflicts of Interest

The authors declare no conflicts of interest.
